# Regional Heterogeneity in Ischemic Heart Disease and Ischemic Stroke Burden in Europe, 1990–2023: GBD 2023 and Complementary Mendelian Randomization Evidence

**DOI:** 10.1002/clc.70505

**Published:** 2026-10-23

**Authors:** Haoyang Hu, Ming Li, Siyuan Zhang, Hong Zhao, Yang Liu, Fei Yang

**Affiliations:** ^1^ The First Medical Center, Chinese PLA General Hospital Beijing China; ^2^ Medical School of Chinese PLA Beijing China; ^3^ Department of Neurology Chinese PAP Force Hospital of Beijing Beijing China

**Keywords:** Europe, Global Burden of Disease, ischemic heart disease, ischemic stroke, Mendelian randomization, risk factors

## Abstract

**Background:**

Ischemic heart disease (IHD) and ischemic stroke (IS) remain major contributors to health loss in Europe, with regional heterogeneity. Comparisons of burden, attributable risks, and genetic evidence remain limited.

**Methods:**

Global Burden of Disease (GBD) 2023 estimates for Central, Eastern, and Western Europe from 1990 to 2023 were analyzed. Age‐standardized prevalence, incidence, mortality, and disability‐adjusted life‐year rates were summarized; temporal trends were assessed by Joinpoint regression and 2023 cross‐country concordance by Spearman correlation. GBD risk profiles were compared between 1990 and 2023. Complementary Mendelian randomization (MR) evaluated high blood pressure, fasting glucose, and low‐density lipoprotein cholesterol (LDL‐C).

**Results:**

Eastern Europe had the highest 2023 burden. IHD and IS rates were positively concordant across countries (Spearman ρ = 0.681–0.820; all *p* < 0.001), and regional 1990–2023 average annual percent changes were negative. High systolic blood pressure and high LDL‐C remained leading GBD‐attributable risks. In MR, genetic liability to high blood pressure was associated with IHD (odds ratio [OR] = 1.354, 95% confidence interval [CI] 1.281–1.433) and IS (OR = 1.351, 95% CI 1.295–1.409), and LDL‐C with IHD (OR = 1.610, 95% CI 1.508–1.719); these met the Bonferroni threshold. Fasting glucose showed nominal evidence for IHD, whereas fasting glucose and LDL‐C showed no statistically significant associations with overall IS.

**Conclusions:**

IHD and IS burdens declined across Europe, but regional inequalities persisted. GBD attribution and MR provide complementary evidence; cardiometabolic risks remain central, while genetic associations differ between IHD and overall IS.

## Graphical abstract

Across Europe, ischemic heart disease and ischemic stroke burdens declined from 1990 to 2023 but remained highest in Eastern Europe. GBD attribution highlighted persistent cardiometabolic risks, while complementary Mendelian randomization supported blood‐pressure‐related associations with both outcomes and LDL cholesterol with ischemic heart disease.

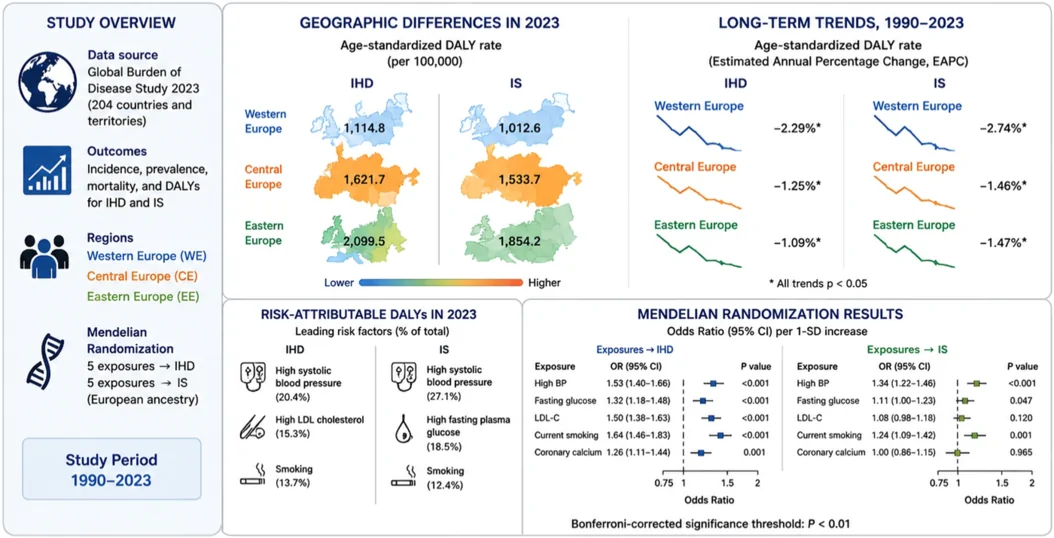

**Abbreviations**
AAPC: average annual percent change
APC: annual percent change
ASDR: age‐standardized disability‐adjusted life‐year rate
ASIR: age‐standardized incidence rate
ASMR: age‐standardized mortality rate
ASPR: age‐standardized prevalence rate
CI: confidence interval
CRA: comparative risk assessment
DALY: disability‐adjusted life year
GBD: Global Burden of Disease
GWAS: genome‐wide association study
HFPG: high fasting plasma glucose
IHD: ischemic heart disease
IS: ischemic stroke
IVW: inverse‐variance weighted
LDL‐C: low‐density lipoprotein cholesterol
MR: Mendelian randomization
SDI: Socio‐demographic Index
SNP: single‐nucleotide polymorphism
UI: uncertainty interval

## 1 Introduction

Ischemic heart disease (IHD) and ischemic stroke (IS) are major contributors to global mortality and disability. Contemporary burden studies show that both diseases continue to account for substantial health loss despite long‐term improvements in prevention and acute care [1, 2, 3]. Their population‐level trajectories are not uniform, and the balance between declining age‐standardized rates and persistent absolute health loss has become increasingly important for health‐system planning [1, 2].

Europe is especially informative for comparative analysis because cardiovascular burden varies markedly across Central, Eastern, and Western European settings. Prior work has described pronounced geographic correspondence between IHD and IS burden and substantial regional variation in associations with established cardiovascular risk factors [4, 5]. These differences may reflect multiple interacting influences, including risk‐factor distributions, socioeconomic conditions, health‐care access, and historical environmental exposures; however, ecological burden data cannot by themselves identify the causal contribution of these factors at the individual level [4, 5, 6].

IHD and IS share several established modifiable risk factors, particularly elevated blood pressure, atherogenic lipids, smoking, diabetes‐related traits, and adverse dietary or environmental exposures [6]. The GBD comparative risk assessment framework quantifies the population burden attributable to such exposures, whereas Mendelian randomization (MR) addresses a different question by using genetic variants as instrumental variables to evaluate exposure–outcome associations under specific assumptions. These approaches therefore provide different and potentially complementary levels of evidence rather than a direct validation framework.

Accordingly, this study aimed to provide an integrated assessment of IHD and IS burden across Europe by characterizing geographic differences in 2023, quantifying long‐term trends in age‐standardized burden from 1990 to 2023, and examining exploratory relationships with the Socio‐demographic Index (SDI) and age‐specific burden patterns. We also assessed temporal changes in GBD‐attributable risk profiles and used Mendelian randomization as a complementary approach to evaluate selected cardiometabolic traits for which large European‐ancestry GWAS datasets and suitable genetic instruments were available.

## 2 Methods

### 2.1 Study Design and GBD Data Sources

We conducted a population‐level observational analysis using estimates from the GBD 2023 framework [7, 8]. Epidemiological estimates for IHD and IS were obtained for the GBD Central Europe, Eastern Europe, and Western Europe regions for 1990–2023. Country‐level estimates in the analytical dataset covered 44 countries or territories across these three regions. GBD data were accessed through the Institute for Health Metrics and Evaluation GBD Results Tool (https://vizhub.healthdata.org/gbd-results/). Reporting of the health‐estimate component was guided by the GATHER principles [9].

We analyzed prevalence, incidence, deaths, and disability‐adjusted life years (DALYs). For regional and temporal comparisons, rates were age‐standardized to the GBD standard population and are reported per 100,000 population with 95% uncertainty intervals (UIs). The corresponding metrics were ASPR, ASIR, ASMR, and ASDR; country‐level estimates for 2023 are provided in Supporting Information S1: Tables 1–4. DALYs combine years of life lost because of premature death and years lived with disability. SDI was used as a composite indicator of socioeconomic development; it was treated as an ecological development indicator and not as a direct measure of health‐care quality or access.

### 2.2 Geographic Comparison and Cross‐Country Concordance

For 2023, country‐level ASPR, ASIR, ASMR, and ASDR were summarized separately for IHD and IS. Because the study did not assess individual‐level coexistence of IHD and IS, we use the terms comparative burden, geographic overlap, and cross‐country concordance rather than comorbidity. To formally quantify population‐level concordance, Spearman rank correlations were calculated between country‐level IHD and IS age‐standardized rates for each metric. Approximate 95% confidence intervals (CIs) for Spearman coefficients were obtained using Fisher z transformation. Regional aggregate rows were excluded from these correlations. Forty‐four countries contributed to analyses of ASPR, ASIR, and ASMR; 43 contributed to ASDR because one country‐level ASDR pair was unavailable in the source supplementary table.

### 2.3 Temporal Trend Analysis

Temporal trends in ASPR, ASIR, ASMR, and ASDR from 1990 to 2023 were analyzed with Joinpoint Regression Program version 5.1.0.0 (National Cancer Institute). Log‐linear segmented models were fitted, and statistically supported changes in slope were identified using Monte Carlo permutation testing with 10 000 permutations. Annual percent change (APC) with 95% CI was estimated for each segment, and average annual percent change (AAPC) with 95% CI summarized the full 1990–2023 period. Statistical significance for individual trend estimates was defined as *p* < 0.05. Detailed APC segments are provided in Supporting Information S1: Tables 5–8; the main text emphasizes AAPCs and selected joinpoints.

### 2.4 Exploratory SDI and Age‐Specific Analyses

SDI was plotted against each age‐standardized burden metric by European region from 1990 to 2023 with a smooth trend curve for exploratory visualization. These plots were treated as descriptive because country‐year observations are serially structured and both SDI and disease burden vary with calendar time; no independent causal effect of SDI was inferred from the graphical relationship, and no unsupported significance statement was used.

Age‐specific numbers and rates for prevalence, incidence, mortality, and DALYs were described across the European population using the age‐group data available in the GBD extraction. The age tables were aggregated for Europe rather than stratified by the three European regions. Accordingly, this analysis described age‐specific distributions rather than regional age decomposition. Detailed age‐specific results are provided in Supporting Information S1: Tables 9–12.

### 2.5 GBD Risk‐Factor Attribution

Attributable burden was assessed using the GBD comparative risk assessment framework [7]. The analysis included all available risk factors for which GBD 2023 provided attributable‐burden estimates for IHD or IS in the European regions examined. Inclusion required an applicable GBD risk–outcome pair and an available outcome‐specific estimate in the regional extraction. Risks were retained separately for each disease, including disease‐specific risks; inclusion did not require a risk to apply to both outcomes. Risks without an applicable outcome‐specific estimate in this extraction were outside the analysis. The complete outcome‐specific inventory, including diet high in trans fatty acids for IHD, is provided in Supporting Information S1: Table 13.

We compared risk‐specific attributable proportions and rankings for DALYs and deaths in 1990 and 2023 across Central, Eastern, and Western Europe. The GBD risk‐factor inventory was independent of GWAS availability, which affected only the complementary MR analysis. Because GBD risk attribution is hierarchical and individual risks may overlap, risk‐specific attributable fractions were not summed as mutually exclusive components.

### 2.6 Mendelian Randomization Data Sources and Exposure Selection

A complementary two‐sample MR analysis was performed and reported in accordance with STROBE‐MR principles [10]. GWAS summary statistics were obtained from the MRC IEU OpenGWAS database and linked source studies. MR focused on genetic liability to high blood pressure, fasting glucose, and LDL‐C. These traits were selected for their cardiovascular relevance and the availability of suitable predominantly European‐ancestry GWAS summary statistics and genetic instruments among the datasets identified for this study. The restricted MR scope reflected the availability of suitable genetic data rather than a restriction of the GBD risk‐factor analysis. MR was not intended to evaluate every GBD risk or validate the complete GBD attribution ranking.

OpenGWAS ebi‐a‐GCST90013966 represents a binary high‐blood‐pressure phenotype derived from UK Biobank data rather than a continuous systolic blood‐pressure GWAS. The linked source study used whole‐genome regression for quantitative and binary UK Biobank traits [11]. The MR exposure is therefore described as genetic liability to high blood pressure, which differs from the GBD high‐systolic‐blood‐pressure risk definition. Fasting glucose data were from ebi‐a‐GCST90002232 [12], LDL‐C data from ebi‐a‐GCST90002412 [13], IHD outcome data from FinnGen (finn‐b‐I9_ISCHHEART) [14], and overall IS data from ebi‐a‐GCST90018864 [15]. Data‐source characteristics are summarized in Supporting Information S1: Table 14.

### 2.7 Genetic Instruments and MR Statistical Analysis

MR interpretation relied on the three core instrumental‐variable assumptions: (1) relevance—the genetic instruments are associated with the exposure; (2) independence—the instruments are not associated with confounders of the exposure–outcome relationship; and (3) exclusion restriction—the instruments affect the outcome only through the exposure under study [10]. Genetic variants were selected at genome‐wide significance (*p* < 5 × 10^−8^) and clumped for linkage disequilibrium at r^2^ < 0.001 within a 10 000‐kb window. The analysis pipeline excluded weak instruments with F‐statistics ≤ 10. Effect alleles were harmonized between exposure and outcome datasets before analysis.

The inverse‐variance weighted (IVW) estimator was the primary MR method, with odds ratios (ORs) and 95% CIs reported for binary outcomes. Weighted‐median and MR‐Egger estimators, together with simple‐ and weighted‐mode estimates, were used as complementary sensitivity approaches. Scatter plots, SNP‐level forest plots, leave‐one‐out analyses, and funnel plots are provided in Supporting Information S1: Figures 1–6. Because six primary exposure–outcome comparisons were evaluated (three exposures × two outcomes), *p* < 0.0083 (0.05/6) was considered strong evidence after Bonferroni correction; *P* values from 0.0083 to < 0.05 were described as nominal evidence and interpreted cautiously. All MR analyses were conducted in R version 4.4.1 using MR packages including TwoSampleMR and MendelianRandomization. Sensitivity was evaluated using the complementary estimators and graphical diagnostics described above.

Potential participant overlap between all contributing exposure and outcome GWAS datasets could not be fully excluded from the publicly available metadata. This possibility, together with the assumptions of two‐sample MR and ancestry restrictions, was considered when interpreting the genetic results.

## 3 Results

### 3.1 Geographic Distribution and Cross‐Country Concordance in 2023

In 2023, Eastern Europe had the highest regional age‐standardized burden for both IHD and IS. For example, the ASIR was 345.62 (95% UI 292.69–406.71) per 100 000 for IHD and 134.08 (116.71–154.42) for IS in Eastern Europe, compared with 131.60 (115.57–148.24) and 60.29 (54.05–66.50), respectively, in Western Europe. Central Europe had intermediate ASIRs of 199.63 (176.12–225.61) for IHD and 103.28 (92.59–113.52) for IS. Similar regional ordering was observed for mortality and DALY rates (Supporting Information S1: Tables 1–4; Figure 1).

**FIGURE 1:**
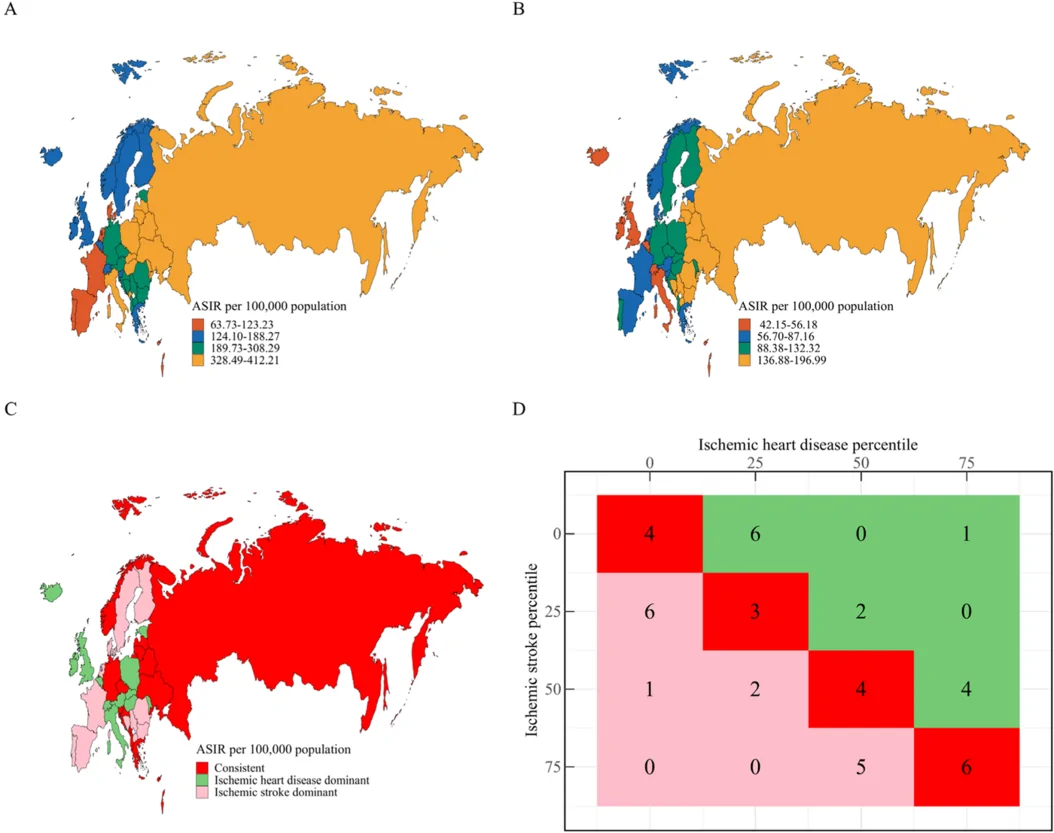
Geographic distribution and comparative burden of ischemic heart disease (IHD) and ischemic stroke (IS) in Europe in 2023. Map panels display age‐standardized incidence patterns, and the country‐level matrix summarizes comparative burden. Spearman correlations between IHD and IS age‐standardized rates are reported in Results Section 3.1 and Supporting Information S1: Table 15. (A) Age‐standardized incidence rate (ASIR) of ischemic heart disease (IHD) per 100,000 population. (B) ASIR of ischemic stroke (IS) per 100,000 population. (C) Country‐level comparison of IHD and IS ASIR percentile categories, classified as consistent, IHD‐dominant, or IS‐dominant. (D) Joint distribution of countries across IHD and IS ASIR percentile categories, with cell values indicating the number of countries.

At the country level, IHD and IS burdens were positively concordant. Spearman correlations were ρ = 0.729 (95% CI 0.552–0.843; *p* < 0.001) for ASIR, ρ = 0.681 (0.482–0.814; *p* < 0.001) for ASPR, ρ = 0.814 (0.682–0.895; *p* < 0.001) for ASMR, and ρ = 0.820 (0.690–0.899; *p* < 0.001) for ASDR (Supporting Information S1: Table 15). These correlations describe population‐level correspondence in country burden and should not be interpreted as individual‐level comorbidity.

Country‐level extremes also varied by metric. Ukraine had the highest IHD ASPR and ASIR in the 2023 data, Belarus had the highest IHD ASMR, and North Macedonia had the highest IS ASIR and ASMR. These estimates and their UIs are reported in Supporting Information S1: Tables 1–4.

### 3.2 Temporal Trends in Age‐Standardized Burden, 1990–2023

All 1990–2023 regional AAPCs were negative for ASPR, ASIR, ASMR, and ASDR for both diseases (Figure 2; Supporting Information S1: Tables 5–8). For IHD, the ASPR AAPC was −1.56% (95% CI − 1.81 to −1.32) in Central Europe, −0.47% (−0.61 to −0.33) in Eastern Europe, and −1.76% (−1.86 to −1.66) in Western Europe. IHD ASMR declined by −2.18% (−2.36 to −2.00), −1.11% (−1.53 to −0.70), and −3.26% (−3.46 to −3.06) per year, respectively. IHD ASDR showed a similar gradient in the magnitude of decline: −2.38% (−2.56 to −2.20), −1.01% (−1.48 to −0.55), and −3.26% (−3.43 to −3.10).

**FIGURE 2:**
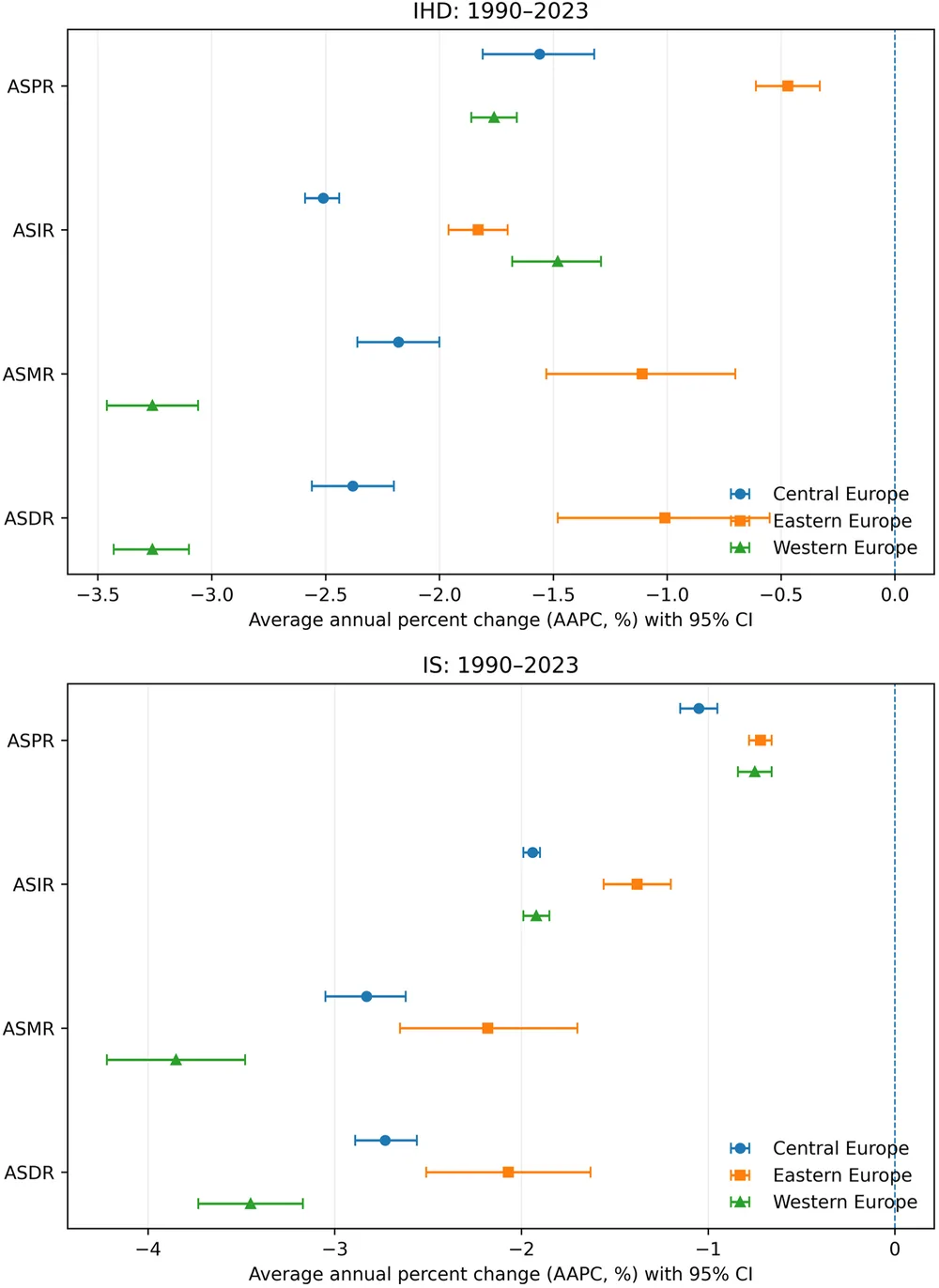
Average annual percent change (AAPC) in age‐standardized IHD and IS burden, 1990–2023. Point estimates and 95% CIs are shown for ASPR, ASIR, ASMR, and ASDR in Central, Eastern, and Western Europe. Detailed joinpoint segments and APC estimates are provided in Supporting Information S1: Tables 5–8 and Supporting Information S1: Figure 7.

For IS, ASPR AAPCs were −1.05% (95% CI − 1.15 to −0.95) in Central Europe, −0.72% (−0.78 to −0.66) in Eastern Europe, and −0.75% (−0.84 to −0.66) in Western Europe. ASIR declined by −1.94% (−1.99 to −1.90), −1.38% (−1.56 to −1.20), and −1.92% (−1.99 to −1.85), respectively. The largest long‐term declines were observed for IS mortality in Western Europe (ASMR AAPC −3.85%, 95% CI − 4.22 to −3.48) and IS DALY rate in Western Europe (ASDR AAPC − 3.45%, −3.73 to −3.17).

Joinpoint segments showed non‐linear trajectories. In Eastern Europe, IHD ASPR increased during 1990–2000 (APC 0.58%, 95% CI 0.30–0.87) and declined thereafter; IHD ASIR also changed across several segments, including a marked decline during 2008–2019. In Western Europe, IHD ASPR increased modestly during 2020–2023 (APC 0.77%, 95% CI 0.22–1.32; *p* = 0.009). Detailed APC estimates and joinpoint periods are provided in Supporting Information S1: Tables 5–8 and Supporting Information S1: Figure 7.

### 3.3 Exploratory Relationship Between SDI and Disease Burden

Exploratory plots showed that higher SDI values generally coincided with lower age‐standardized IHD and IS burden, especially for mortality and DALY rates. Eastern European observations often remained at higher burden levels over comparable portions of the SDI range (Figure 3). These temporally structured trajectories were interpreted descriptively.

**FIGURE 3:**
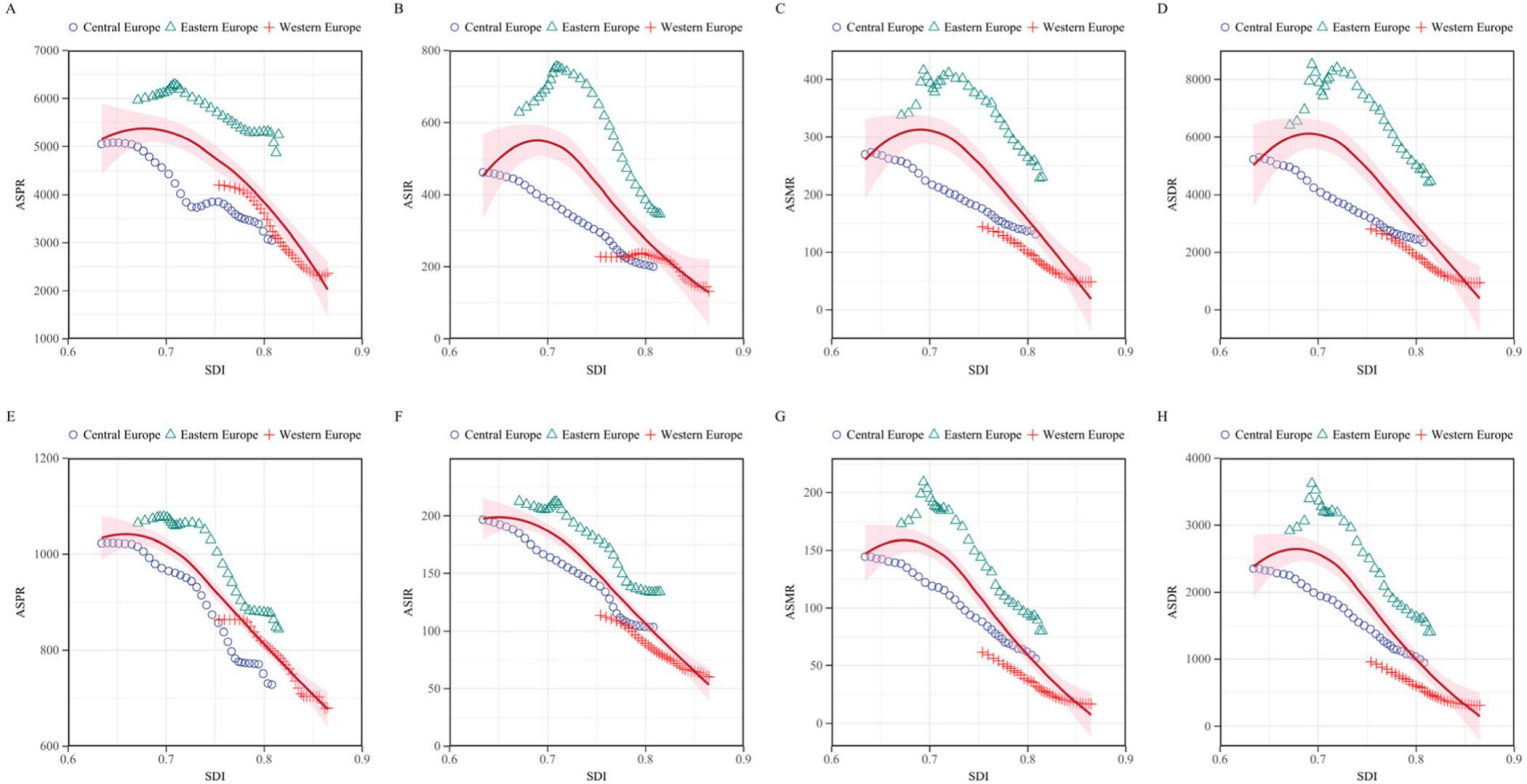
Exploratory relationship between Socio‐demographic Index (SDI) and age‐standardized IHD and IS burden in Central, Eastern, and Western Europe, 1990–2023. Curves describe regional trajectories across SDI values and are interpreted as ecological patterns. Panels (A–D) show the relationships between the Socio‐demographic Index (SDI) and age‐standardized prevalence, incidence, mortality, and disability‐adjusted life‐year (DALY) rates, respectively, for ischemic heart disease (IHD). Panels (E–H) show the corresponding relationships for ischemic stroke (IS), in the same order. All rates are expressed per 100,000 population.

### 3.4 Age‐Specific Distribution of IHD and IS Burden

Age‐specific patterns demonstrated increasing rates with advancing age, but the pattern differed between counts and rates (Supporting Information S1: Tables 9–12 and Supporting Information S1: Figure 8). IHD prevalence count peaked at ages 65–69, whereas the prevalence rate peaked at ages 85–89 and was slightly lower thereafter. IS prevalence count peaked at ages 70–74, while the prevalence rate continued to increase through the oldest age group. Incidence rates for both IHD and IS increased progressively and were highest in the 95+ group in the available data.

Mortality rates and DALY rates also increased steeply with age for both diseases and were highest in the 95+ group. In contrast, absolute death counts peaked at ages 85–89 for both IHD and IS, and DALY counts peaked at ages 70–74 for IHD and 80–84 for IS. These findings describe the age‐specific concentration of burden.

### 3.5 Changes in Attributable Risk‐Factor Profiles

High systolic blood pressure and high LDL‐C remained leading GBD‐attributable risks for IHD and IS across European regions in both 1990 and 2023 (Figure 4). Changes were most apparent among lower‐ranked risks, alongside the persistence of leading cardiometabolic risks.

**FIGURE 4:**
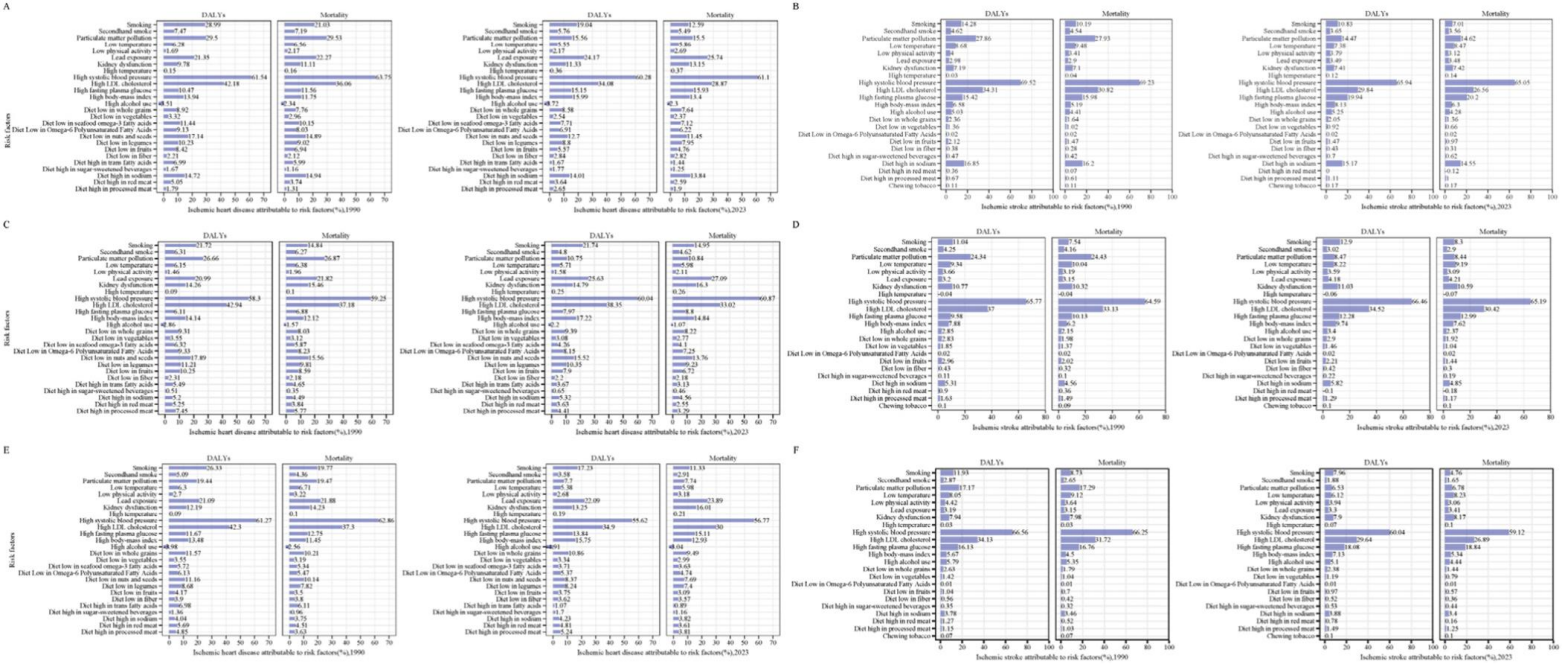
GBD 2023 risk‐factor attribution profiles for IHD and IS in Europe in 1990 and 2023. Panels show IHD and IS, respectively, in Central Europe (A, B), Western Europe (C, D), and Eastern Europe (E, F). Within each panel, DALY and mortality attributable fractions are displayed for 1990 on the left and 2023 on the right. Risk factors were included separately for each outcome according to GBD availability; the inventory is provided in Supporting Information S1: Table 13. Risk‐specific attributable fractions may overlap and are not additive.

For IHD, lead exposure increased in relative rank among the displayed environmental risks. In Central Europe in 2023, lead exposure accounted for 24.17% of IHD DALYs and 25.74% of IHD mortality in the GBD attribution estimates; in Eastern Europe, the corresponding DALY fraction was 25.63%. For IS, the relative importance of HFPG increased in the displayed profile: in Central Europe, HFPG accounted for 19.94% of IS DALYs and 20.20% of IS mortality in 2023; in Eastern Europe, HFPG accounted for 12.99% of IS mortality. In Western Europe, HFPG accounted for 18.08% of IS DALYs in 2023. These risk‐specific fractions may overlap and are not additive.

### 3.6 Complementary Mendelian Randomization Analysis

In the primary IVW analysis, genetic liability to high blood pressure was associated with higher odds of IHD (OR = 1.354, 95% CI 1.281–1.433, *p* < 0.001; 184 SNPs) and overall IS (OR = 1.351, 95% CI 1.295–1.409, *p* < 0.001; 201 SNPs) (Figure 5). Higher genetically predicted LDL‐C was associated with IHD (OR = 1.610, 95% CI 1.508–1.719, *p* < 0.001; 321 SNPs). These three associations met the Bonferroni‐corrected threshold of *p* < 0.0083.

**FIGURE 5:**
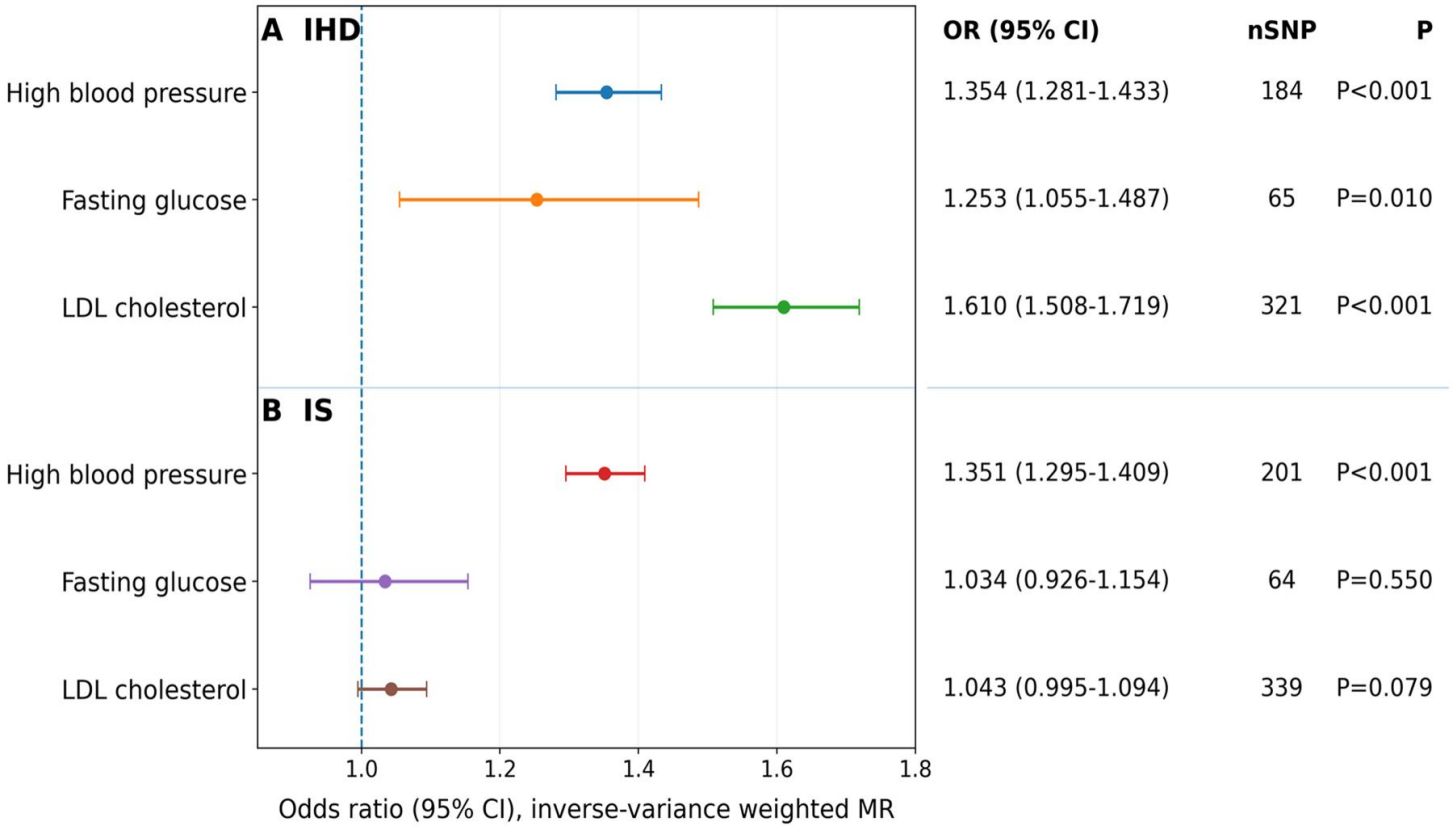
Complementary two‐sample Mendelian randomization estimates for selected cardiometabolic traits and (A) IHD and (B) overall IS. The primary estimator was inverse‐variance weighted MR. The blood‐pressure exposure represents genetic liability to high blood pressure (OpenGWAS ebi‐a‐GCST90013966). ORs and 95% CIs are shown alongside instrument counts and *p* values. *p* < 0.0083 was the Bonferroni‐corrected threshold for six primary comparisons.

Fasting glucose showed nominal evidence of association with IHD (OR = 1.253, 95% CI 1.055–1.487, *p* = 0.010; 65 SNPs), but this result did not meet the Bonferroni‐corrected threshold. For overall IS, neither fasting glucose (OR = 1.034, 95% CI 0.926–1.154, *p* = 0.550; 64 SNPs) nor LDL‐C (OR = 1.043, 95% CI 0.995–1.094, *p* = 0.079; 339 SNPs) reached *p* < 0.05. The LDL‐C estimate for IS was close to the null boundary, and the absence of statistical significance should not be interpreted as evidence that dyslipidemia is irrelevant to all ischemic stroke mechanisms or subtypes.

Sensitivity estimators and diagnostic plots are presented in Supporting Information S1: Figures 1–6. They include MR‐Egger, weighted‐median, simple‐mode and weighted‐mode estimates, SNP‐level forest plots, leave‐one‐out analyses, and funnel plots.

## 4 Discussion

IHD and IS burden declined across Central, Eastern, and Western Europe, but substantial geographic inequalities persisted. Eastern Europe retained the highest age‐standardized burden, and country‐level concordance was strongest for mortality and DALY rates. GBD attribution identified continuing cardiometabolic priorities alongside regional variation in environmental and dietary risks. Complementary MR supported associations of genetic liability to high blood pressure with both outcomes and LDL‐C with IHD. Together, these findings indicate overlapping cardiovascular risk pathways with disease‐specific patterns of genetic evidence.

The geographic concordance is consistent with earlier evidence of East–West differences in cardiovascular burden and risk‐factor associations [4, 5, 16]. The correspondence in mortality and DALY rates may reflect common exposure patterns and differences in prevention, acute care, and long‐term disease management. Persistent regional inequalities therefore provide context for interpreting the two diseases together while retaining their distinct clinical characteristics.

Long‐term declines in age‐standardized burden are consistent with previous IHD and IS studies [1, 2, 3, 17]. The slower improvement in several Eastern European IHD indicators suggests that gains have been uneven across regions. Meanwhile, the concentration of age‐specific burden in older adults highlights continuing demand for cardiovascular prevention and care. Declining standardized rates and substantial burden in older populations represent complementary dimensions of the European cardiovascular challenge.

The inverse ecological pattern between SDI and disease burden places these regional inequalities within a broader development context. Differences in risk‐factor control, medication access, and health‐care delivery may contribute to the observed trajectories [5, 16]. The higher burden in Eastern Europe across comparable portions of the SDI range suggests that development level alone does not fully describe regional variation.

The persistence of high systolic blood pressure and high LDL‐C in the GBD profiles reinforces their importance alongside other established cardiovascular risks [6, 7, 18, 19, 20]. Lead exposure remained prominent within the IHD environmental profile, consistent with its recognized cardiovascular toxicity [21]. The greater relative contribution of HFPG to IS burden adds a metabolic dimension to regional prevention priorities. Dietary, pollution, and temperature‐related burdens further emphasize that cardiovascular health depends on multiple exposure pathways [7, 22, 23, 24]. These profiles support a broad prevention perspective alongside sustained attention to leading cardiometabolic risks.

IHD commonly involves coronary atherosclerosis, whereas IS encompasses large‐artery atherosclerosis, small‐vessel occlusion, and cardioembolic mechanisms [6, 25]. Elevated blood pressure can affect both coronary and cerebral vascular beds through vascular injury and remodeling. In cerebral small vessels, sustained pressure‐related damage offers a pathway to occlusion and ischemic injury. Previous MR evidence links higher blood pressure to the major IS subtypes, with stronger associations for large‐artery and small‐vessel stroke than cardioembolic stroke [26]. This broad vascular involvement provides biological context for the blood‐pressure associations observed with both outcomes in our analysis.

LDL‐C is closely related to atherosclerotic disease, making its association with IHD biologically coherent [6]. Its contribution to IS may vary with the mixture of underlying stroke mechanisms. Prior MR studies support an association with large‐artery atherosclerotic stroke and report weaker genetic associations with overall IS than coronary disease [27, 28]. Combining atherosclerotic, small‐vessel, and cardioembolic events can therefore obscure associations concentrated in particular subtypes. Our LDL‐C result for overall IS is compatible with a small effect, and subtype composition offers a plausible interpretation of the observed pattern. It does not establish that lipid‐related pathways are unimportant in IS.

Glycemic phenotypes also require disease‐specific interpretation. Clinical diabetes combines hyperglycemia with insulin resistance, beta‐cell dysfunction, and other metabolic disturbances. A fasting‐glucose genetic proxy captures only part of this phenotype. Previous MR work found associations of type 2 diabetes and higher HbA1c with large‐artery and small‐vessel stroke, while fasting‐glucose findings differed [29]. This distinction helps contextualize the GBD HFPG burden alongside the weaker fasting‐glucose evidence in our MR analysis. The fasting‐glucose association with IHD was nominally significant but did not meet the multiple‐comparison threshold. Neither that result nor the overall IS estimate should be generalized to all diabetic vascular pathways.

GBD and MR describe different dimensions of risk. GBD attributable burden reflects exposure distributions, outcome burden, and the comparative‐risk model, whereas MR evaluates genetically proxied exposure–outcome associations. The binary high‐blood‐pressure GWAS phenotype also differs from the GBD continuous systolic‐blood‐pressure risk definition. Their combined interpretation can identify convergent priorities without requiring identical rankings or numerical estimates. Differences between the GBD and MR patterns may therefore reflect exposure definitions and population burden as well as disease biology.

The study combines contemporary GBD estimates, country‐level concordance, temporal trends, and complementary genetic evidence within a European regional comparison. Outcome‐specific risk inventories and clearly defined GWAS phenotypes make the scope of these complementary analyses transparent.

Several limitations remain. GBD estimates depend on source‐data quality and modeling, which vary across locations; UIs accompany the estimates [7, 8, 9]. Country‐level correlations and SDI trajectories are ecological and cannot establish individual‐level relationships or explain regional determinants. GBD risk‐specific fractions may overlap. Age‐specific data were aggregated for Europe and did not support regional age decomposition. MR covered three selected traits and relied on assumptions vulnerable to horizontal pleiotropy, population structure, and potential sample overlap. Overall IS combined etiologic subtypes, which may obscure subtype‐specific associations. Sensitivity assessment relied on alternative estimators and graphical diagnostics. Numerical heterogeneity and directional‐pleiotropy statistics were not reported, limiting the assessment of robustness. A difference in statistical significance between outcomes does not itself demonstrate a difference in effects.

The findings support sustained attention to established cardiometabolic risk control alongside environmental and dietary risks. Persistent inequalities, particularly in Eastern Europe, and the concentration of burden in older adults highlight complementary priorities for population prevention, clinical care, and disease‐specific risk assessment.

## 5 Conclusions

From 1990 to 2023, age‐standardized IHD and IS burdens generally declined across Central, Eastern, and Western Europe, while substantial regional inequalities persisted. The two diseases showed strong country‐level concordance in 2023. GBD attribution identified persistent cardiometabolic risks alongside region‐specific environmental and dietary profiles. Complementary MR supported associations of genetic liability to high blood pressure with both outcomes and LDL‐C with IHD, whereas evidence for fasting glucose and LDL‐C in overall IS was less robust. These patterns support a joint cardiovascular prevention perspective that also recognizes the heterogeneous mechanisms underlying IS.

## Author Contributions

Study concept and design: Haoyang Hu, Ming Li, and Fei Yang. Acquisition of data: Haoyang Hu, Ming Li, Hong Zhao, and Yang Liu. Data analysis and manuscript drafting: Haoyang Hu and Siyuan Zhang. Review, editing, and supervision: Fei Yang. All authors contributed intellectual content, reviewed the revised manuscript, and approved the final version. Haoyang Hu, Ming Li, and Siyuan Zhang contributed equally.

## Funding

The authors have nothing to report.

## Ethics Statement

This study used publicly available, aggregated GBD estimates and de‐identified GWAS summary statistics. The requirement for additional ethical approval was waived by the Ethics Committee of The First Medical Center, Chinese PLA General Hospital. Original GWAS studies obtained approvals and participant consent under their respective protocols.

## Consent

The authors have nothing to report.

## Conflicts of Interest

The authors declare no conflicts of interest.

## Supporting information


Supporting File 1

Supporting File 2

## Data Availability

The data that support the findings of this study are available from the corresponding author upon reasonable request. GBD 2023 estimates can be accessed through the IHME GBD Results Tool (https://vizhub.healthdata.org/gbd-results/). GWAS summary statistics are available through the MRC IEU OpenGWAS project (https://opengwas.io/), including ebi‐a‐GCST90013966, ebi‐a‐GCST90002232, ebi‐a‐GCST90002412, finn‐b‐I9_ISCHHEART, and ebi‐a‐GCST90018864. Analytical values underlying the cross‐country correlations were taken from Supporting Information S1: Tables 1–4.
