# Supplementary material for: Regional Heterogeneity in Ischemic Heart Disease and Ischemic Stroke Burden in Europe, 1990–2023: GBD 2023 and Complementary Mendelian Randomization Evidence
Source: Clin Cardiol. 2026 Oct 23;49(10):e70505. doi: 10.1002/clc.70505 (PMC13691926; doi:10.1002/clc.70505)
Supplement: Supplementary file 1 [file CLC-49-e70505-s001.docx]

**Supplementary Table 1 Incidence of Ischemic heart disease and Ischemic stroke by country in Europe, 2023**

| Location | | | Ischemic heart disease | | Ischemic stroke | |
| --- | --- | --- | --- | --- | --- | --- |
|  |  |  | Incidence number(95%UI) | ASIR (95%UI) per 100,000 | Incidence number(95%UI) | ASIR (95%UI) per 100,000 |
|  | Central Europe |  | 451933 ( 398389 , 515558 ) | 199.63 ( 176.12 , 225.61 ) | 235435 ( 208823 , 261697 ) | 103.28 ( 92.59 , 113.52 ) |
|  |  | Albania | 8829 ( 7692 , 9997 ) | 201.27 ( 176.43 , 227.65 ) | 3230 ( 2769 , 3843 ) | 76.07 ( 65.89 , 89.63 ) |
|  |  | Bosnia and Herzegovina | 11612 ( 9978 , 13301 ) | 188.28 ( 162.57 , 214.62 ) | 8478 ( 7322 , 9875 ) | 137.84 ( 119.8 , 157.84 ) |
|  |  | Bulgaria | 29862 ( 26324 , 34157 ) | 203.12 ( 179.47 , 230.67 ) | 25602 ( 22598 , 28848 ) | 173.65 ( 155.59 , 191.52 ) |
|  |  | Croatia | 14774 ( 12966 , 16956 ) | 169.02 ( 148.8 , 191.86 ) | 7617 ( 6754 , 8454 ) | 84.36 ( 75.02 , 93.11 ) |
|  |  | Czechia | 39928 ( 35148 , 45938 ) | 182.8 ( 159.52 , 206.28 ) | 15095 ( 13400 , 17177 ) | 70.2 ( 61.93 , 79.09 ) |
|  |  | Hungary | 43132 ( 37761 , 49605 ) | 219.54 ( 193.03 , 250.04 ) | 18354 ( 16032 , 21135 ) | 95 ( 83.81 , 107.2 ) |
|  |  | Montenegro | 2368 ( 2026 , 2717 ) | 216.28 ( 186.63 , 247.72 ) | 1305 ( 1113 , 1531 ) | 118.99 ( 102.73 , 137.56 ) |
|  |  | North Macedonia | 5893 ( 5078 , 6728 ) | 180.97 ( 157.24 , 207.31 ) | 6512 ( 5125 , 7835 ) | 196.99 ( 159.21 , 232.53 ) |
|  |  | Poland | 160869 ( 139854 , 185187 ) | 216.76 ( 188.21 , 248.25 ) | 65245 ( 58147 , 73316 ) | 87.17 ( 78.44 , 97.75 ) |
|  |  | Romania | 71245 ( 62773 , 80630 ) | 189.73 ( 167.77 , 212.94 ) | 49880 ( 42774 , 58181 ) | 130.01 ( 113.67 , 148.16 ) |
|  |  | Serbia | 30602 ( 26022 , 35819 ) | 188.16 ( 162.26 , 217.21 ) | 19201 ( 16119 , 22809 ) | 119.22 ( 101.67 , 137.65 ) |
|  |  | Slovakia | 20443 ( 17732 , 23874 ) | 210.6 ( 183.97 , 243.81 ) | 8681 ( 7582 , 9948 ) | 90.73 ( 79.82 , 102.62 ) |
|  |  | Slovenia | 5614 ( 4823 , 6507 ) | 123.23 ( 106.33 , 141.79 ) | 2714 ( 2390 , 3054 ) | 56.19 ( 49.64 , 63.86 ) |
|  | Eastern Europe |  | 1255971 ( 1062429 , 1495533 ) | 345.62 ( 292.69 , 406.71 ) | 473531 ( 408581 , 552579 ) | 134.08 ( 116.71 , 154.42 ) |
|  |  | Belarus | 62972 ( 55223 , 72109 ) | 379.84 ( 335.47 , 431.01 ) | 19109 ( 16830 , 21956 ) | 117.41 ( 104.24 , 132.01 ) |
|  |  | Estonia | 4188 ( 3660 , 4807 ) | 144.66 ( 126.32 , 165.31 ) | 1794 ( 1596 , 2034 ) | 64.7 ( 57.16 , 73.43 ) |
|  |  | Latvia | 9655 ( 8329 , 11024 ) | 232.14 ( 199.94 , 264.65 ) | 5705 ( 5099 , 6406 ) | 132.33 ( 118.6 , 148.07 ) |
|  |  | Lithuania | 17528 ( 15492 , 19911 ) | 282.82 ( 249.47 , 320.55 ) | 6545 ( 5777 , 7368 ) | 108.38 ( 96.38 , 121.12 ) |
|  |  | Republic of Moldova | 19563 ( 17001 , 22259 ) | 308.29 ( 268.04 , 351.54 ) | 6510 ( 5650 , 7452 ) | 104.74 ( 91.48 , 118.47 ) |
|  |  | Russian Federation | 817293 ( 686537 , 981223 ) | 328.5 ( 276.13 , 391.5 ) | 329715 ( 282385 , 385607 ) | 136.88 ( 118.36 , 158.2 ) |
|  |  | Ukraine | 324771 ( 270594 , 386246 ) | 412.21 ( 344.73 , 484.43 ) | 104153 ( 88489 , 123589 ) | 137.55 ( 117.97 , 160.73 ) |
|  | Western Europe |  | 1272633 ( 1121718 , 1432058 ) | 131.6 ( 115.57 , 148.24 ) | 583709 ( 524472 , 638940 ) | 60.29 ( 54.05 , 66.5 ) |
|  |  | Andorra | 163 ( 140 , 188 ) | 92.59 ( 78.44 , 108.23 ) | 82 ( 72 , 95 ) | 47.93 ( 41.5 , 56.19 ) |
|  |  | Austria | 30856 ( 27029 , 35466 ) | 161.05 ( 141.96 , 183.14 ) | 11837 ( 10214 , 13279 ) | 64.74 ( 55.01 , 73.55 ) |
|  |  | Belgium | 24069 ( 21068 , 27569 ) | 100.44 ( 88.12 , 114.96 ) | 11872 ( 10266 , 13676 ) | 49.3 ( 42.54 , 56.69 ) |
|  |  | Cyprus | 1898 ( 1630 , 2183 ) | 96.84 ( 82.62 , 111.07 ) | 998 ( 847 , 1174 ) | 51.26 ( 44.24 , 59.49 ) |
|  |  | Denmark | 10214 ( 9119 , 11509 ) | 85.72 ( 76.84 , 95.6 ) | 6508 ( 5613 , 7406 ) | 53.72 ( 45.98 , 61.39 ) |
|  |  | Finland | 18467 ( 16062 , 21147 ) | 137.64 ( 120.01 , 156.1 ) | 8771 ( 7912 , 9806 ) | 67.81 ( 61.09 , 75.13 ) |
|  |  | France | 111077 ( 98030 , 126748 ) | 74.85 ( 65.44 , 85.27 ) | 75000 ( 66737 , 82427 ) | 52.33 ( 46.04 , 58.2 ) |
|  |  | Germany | 362984 ( 324595 , 407721 ) | 183.26 ( 163.16 , 206.05 ) | 170890 ( 152462 , 189424 ) | 88.38 ( 78.5 , 97.84 ) |
|  |  | Greece | 33006 ( 28819 , 37573 ) | 131.16 ( 115.43 , 148.72 ) | 17682 ( 15497 , 20042 ) | 64.72 ( 56.28 , 73.02 ) |
|  |  | Iceland | 811 ( 702 , 922 ) | 132.22 ( 114.17 , 150.72 ) | 294 ( 251 , 337 ) | 48.84 ( 41.99 , 55.55 ) |
|  |  | Ireland | 10344 ( 9017 , 11693 ) | 120.25 ( 105.89 , 135.59 ) | 3532 ( 3074 , 4065 ) | 42.16 ( 36.63 , 48.48 ) |
|  |  | Israel | 11470 ( 10024 , 13272 ) | 88.12 ( 76.82 , 102.09 ) | 5935 ( 5126 , 6757 ) | 46.47 ( 39.82 , 52.94 ) |
|  |  | Italy | 310612 ( 264776 , 359345 ) | 215.08 ( 185.29 , 247.95 ) | 75091 ( 65397 , 86522 ) | 48.77 ( 42.93 , 55.66 ) |
|  |  | Luxembourg | 951 ( 822 , 1090 ) | 87.62 ( 75.99 , 100 ) | 483 ( 422 , 542 ) | 45.18 ( 39.39 , 50.77 ) |
|  |  | Malta | 1245 ( 1090 , 1441 ) | 121.05 ( 105.48 , 137.24 ) | 460 ( 398 , 527 ) | 45.44 ( 39.2 , 51.74 ) |
|  |  | Monaco | 80 ( 65 , 100 ) | 78.2 ( 63.65 , 97.17 ) | 63 ( 54 , 76 ) | 61.28 ( 53.02 , 71.81 ) |
|  |  | Netherlands | 31858 ( 27373 , 36988 ) | 86.48 ( 73.65 , 99.78 ) | 21386 ( 18216 , 24742 ) | 59.41 ( 50.33 , 67.88 ) |
|  |  | Norway | 10204 ( 8498 , 12137 ) | 97.36 ( 81.97 , 115.62 ) | 5750 ( 4899 , 6776 ) | 56.7 ( 49 , 66.77 ) |
|  |  | Portugal | 19070 ( 16660 , 21878 ) | 76.09 ( 66.12 , 86.7 ) | 18505 ( 16413 , 20550 ) | 69.22 ( 61.71 , 76.58 ) |
|  |  | San Marino | 79 ( 68 , 92 ) | 95.58 ( 81.67 , 109.91 ) | 55 ( 47 , 62 ) | 66.62 ( 57.5 , 75.46 ) |
|  |  | Spain | 65509 ( 57743 , 72551 ) | 63.73 ( 55.47 , 70.95 ) | 56064 ( 49861 , 61500 ) | 54.01 ( 47.59 , 59.39 ) |
|  |  | Sweden | 28909 ( 28209 , 29647 ) | 128.27 ( 125.63 , 131.09 ) | 17608 ( 15356 , 19326 ) | 75.78 ( 66.96 , 82.48 ) |
|  |  | Switzerland | 19380 ( 17047 , 22300 ) | 100.14 ( 88.02 , 114.94 ) | 8013 ( 6892 , 9237 ) | 42.64 ( 36.36 , 48.7 ) |
|  |  | United Kingdom | 168268 ( 141782 , 199300 ) | 124.11 ( 104.88 , 147.93 ) | 66322 ( 56995 , 76567 ) | 48.4 ( 41.97 , 56.07 ) |

**Supplementary Table 2 Prevalence of Ischemic heart disease and Ischemic stroke by country in Europe, 2023**

| **Location** | | | **Ischemic heart disease** | | **Ischemic stroke** | |
| --- | --- | --- | --- | --- | --- | --- |
|  |  |  | **Prevalence number(95%UI)** | **ASPR (95%UI) per 100,000** | **Prevalence number(95%UI)** | **ASPR (95%UI) per 100,000** |
|  | **Central Europe** |  | 6713694 ( 6087999 , 7571366 ) | 3049.61 ( 2775.04 , 3423.83 ) | 1592140 ( 1487461 , 1736480 ) | 728.49 ( 683.43 , 788.41 ) |
|  |  | Albania | 119727 ( 99858 , 141260 ) | 2696.07 ( 2266.31 , 3149.63 ) | 23317 ( 21568 , 24995 ) | 548.78 ( 508.14 , 588.29 ) |
|  |  | Bosnia and Herzegovina | 176942 ( 149917 , 206610 ) | 2911 ( 2488.75 , 3369.95 ) | 55065 ( 51744 , 58125 ) | 914.32 ( 863.38 , 963.97 ) |
|  |  | Bulgaria | 595152 ( 531818 , 661375 ) | 4108.56 ( 3755.22 , 4536.19 ) | 171663 ( 157045 , 196490 ) | 1168.33 ( 1076.62 , 1316.46 ) |
|  |  | Croatia | 198244 ( 166865 , 247489 ) | 2392.33 ( 2045.9 , 2929.3 ) | 53235 ( 50468 , 56270 ) | 627.5 ( 597.13 , 657.96 ) |
|  |  | Czechia | 622383 ( 525845 , 760569 ) | 2972.52 ( 2510.18 , 3595.22 ) | 128749 ( 122418 , 136198 ) | 616.66 ( 586.5 , 650.1 ) |
|  |  | Hungary | 780884 ( 712091 , 905976 ) | 4175.76 ( 3792.85 , 4784.63 ) | 139255 ( 131424 , 147692 ) | 749.83 ( 712.95 , 791.11 ) |
|  |  | Montenegro | 32222 ( 26719 , 37229 ) | 2953.3 ( 2484 , 3390.88 ) | 7871 ( 7294 , 8393 ) | 722.13 ( 671.25 , 769.43 ) |
|  |  | North Macedonia | 82231 ( 71158 , 95188 ) | 2570.79 ( 2231.9 , 2972.33 ) | 35205 ( 33297 , 37378 ) | 1072.71 ( 1012.24 , 1135.02 ) |
|  |  | Poland | 2261603 ( 1862719 , 2717766 ) | 3099.32 ( 2576.05 , 3711.69 ) | 473157 ( 419092 , 544524 ) | 661.9 ( 589.86 , 752.38 ) |
|  |  | Romania | 1004139 ( 892164 , 1219865 ) | 2791.66 ( 2494.99 , 3357.56 ) | 270958 ( 249566 , 290763 ) | 742.65 ( 690.84 , 792.73 ) |
|  |  | Serbia | 400541 ( 337659 , 469158 ) | 2440.07 ( 2078.55 , 2825.49 ) | 121180 ( 113315 , 128717 ) | 748 ( 701.75 , 793.69 ) |
|  |  | Slovakia | 260385 ( 232529 , 300529 ) | 2706.14 ( 2428.29 , 3089.29 ) | 69631 ( 66085 , 73452 ) | 733.09 ( 696.21 , 771.45 ) |
|  |  | Slovenia | 78806 ( 66168 , 96207 ) | 1795.02 ( 1522.72 , 2178.09 ) | 19035 ( 17793 , 20210 ) | 436.81 ( 408.94 , 462.23 ) |
|  | **Eastern Europe** |  | 18954352 ( 16066280 , 22353227 ) | 5248.78 ( 4461.28 , 6197.13 ) | 2953181 ( 2682601 , 3320858 ) | 844.56 ( 769.2 , 943.47 ) |
|  |  | Belarus | 911765 ( 830152 , 1006949 ) | 5598.24 ( 5119.67 , 6134.08 ) | 128479 ( 120534 , 138297 ) | 807.37 ( 760.42 , 865.92 ) |
|  |  | Estonia | 98685 ( 87912 , 117977 ) | 3696.38 ( 3313.69 , 4391.59 ) | 14598 ( 13739 , 15561 ) | 581.41 ( 551.04 , 616.58 ) |
|  |  | Latvia | 123643 ( 109597 , 140364 ) | 3285.44 ( 2935.56 , 3677.68 ) | 28958 ( 27173 , 30763 ) | 784.81 ( 742.03 , 833.04 ) |
|  |  | Lithuania | 288794 ( 256411 , 334352 ) | 5100.4 ( 4558.67 , 5876.74 ) | 44192 ( 41664 , 46678 ) | 808.7 ( 763.43 , 851.42 ) |
|  |  | Republic of Moldova | 347101 ( 305918 , 404479 ) | 5657.11 ( 5002.16 , 6532.49 ) | 43051 ( 40553 , 45765 ) | 725.88 ( 685.63 , 771.39 ) |
|  |  | Russian Federation | 12134194 ( 10102594 , 14703776 ) | 4865.5 ( 4066.19 , 5839.72 ) | 2056916 ( 1850962 , 2313390 ) | 842.47 ( 761.27 , 941.47 ) |
|  |  | Ukraine | 5050171 ( 4202628 , 5980959 ) | 6538.83 ( 5473.09 , 7764.55 ) | 636988 ( 560921 , 735703 ) | 881.84 ( 784.22 , 1004.81 ) |
|  | **Western Europe** |  | 21081583 ( 19349517 , 23654568 ) | 2354.89 ( 2173.09 , 2627.4 ) | 6146379 ( 5843793 , 6448432 ) | 679.05 ( 648.95 , 711.16 ) |
|  |  | Andorra | 2614 ( 2230 , 3041 ) | 1578.47 ( 1340.7 , 1833.63 ) | 882 ( 827 , 938 ) | 553.95 ( 519.48 , 585.59 ) |
|  |  | Austria | 600066 ( 565211 , 658788 ) | 3373.61 ( 3191.78 , 3677.93 ) | 141858 ( 135818 , 147890 ) | 797.22 ( 762.86 , 832.08 ) |
|  |  | Belgium | 393156 ( 352780 , 524477 ) | 1756.31 ( 1584.73 , 2292.89 ) | 120067 ( 114327 , 126051 ) | 545.03 ( 520.59 , 573.99 ) |
|  |  | Cyprus | 31387 ( 26520 , 36504 ) | 1617.16 ( 1367.12 , 1875.89 ) | 8937 ( 8449 , 9464 ) | 467.38 ( 441.31 , 494.26 ) |
|  |  | Denmark | 269999 ( 239019 , 314233 ) | 2336.17 ( 2088.39 , 2678.12 ) | 64964 ( 62058 , 68705 ) | 575.93 ( 548.16 , 607.77 ) |
|  |  | Finland | 381839 ( 329913 , 442213 ) | 3134.71 ( 2729.94 , 3604.29 ) | 85330 ( 81206 , 89910 ) | 723 ( 690.26 , 759.76 ) |
|  |  | France | 2640437 ( 2366002 , 3163090 ) | 1917.51 ( 1719.29 , 2265.9 ) | 820601 ( 785026 , 857767 ) | 603.09 ( 578.51 , 628.3 ) |
|  |  | Germany | 5903701 ( 5420281 , 6471015 ) | 3226.57 ( 3003.36 , 3541.03 ) | 1893308 ( 1836718 , 1963262 ) | 1008.08 ( 977.8 , 1045.74 ) |
|  |  | Greece | 628640 ( 560392 , 750191 ) | 2783.2 ( 2529.81 , 3290.17 ) | 150062 ( 141270 , 157711 ) | 639.39 ( 602.24 , 671.62 ) |
|  |  | Iceland | 10804 ( 9364 , 12879 ) | 1834.89 ( 1612.32 , 2197.71 ) | 3142 ( 2971 , 3305 ) | 544.38 ( 512.64 , 576.41 ) |
|  |  | Ireland | 166243 ( 140030 , 191037 ) | 1993.73 ( 1701.45 , 2275.58 ) | 35507 ( 33274 , 37743 ) | 441.99 ( 414.58 , 468.23 ) |
|  |  | Israel | 217166 ( 196271 , 246685 ) | 1747.24 ( 1589.5 , 1968.38 ) | 71000 ( 67179 , 75111 ) | 578.52 ( 545.49 , 612.39 ) |
|  |  | Italy | 3007823 ( 2571268 , 3505671 ) | 2300.8 ( 1976.68 , 2649.9 ) | 661417 ( 574943 , 743712 ) | 496.87 ( 441.4 , 555.6 ) |
|  |  | Luxembourg | 16491 ( 15122 , 17938 ) | 1555.58 ( 1422.93 , 1688.81 ) | 4770 ( 4536 , 4995 ) | 470.17 ( 446.79 , 492.76 ) |
|  |  | Malta | 19297 ( 17247 , 22576 ) | 1958.53 ( 1778.51 , 2303.68 ) | 4417 ( 4205 , 4663 ) | 457.43 ( 432.94 , 483.16 ) |
|  |  | Monaco | 1337 ( 1105 , 1595 ) | 1355.3 ( 1127.49 , 1622.41 ) | 590 ( 552 , 627 ) | 629.09 ( 590.96 , 663.82 ) |
|  |  | Netherlands | 545567 ( 452438 , 714128 ) | 1559.08 ( 1290.63 , 2021.23 ) | 210537 ( 198548 , 222597 ) | 625.87 ( 593.61 , 660.89 ) |
|  |  | Norway | 308858 ( 257226 , 365005 ) | 3082.18 ( 2559.83 , 3684.93 ) | 68968 ( 58948 , 78162 ) | 702.14 ( 611.64 , 789.29 ) |
|  |  | Portugal | 461372 ( 406076 , 557648 ) | 1995.02 ( 1748.01 , 2381.08 ) | 156751 ( 149154 , 164562 ) | 674.35 ( 641.08 , 707.43 ) |
|  |  | San Marino | 1303 ( 1079 , 1531 ) | 1655.85 ( 1385.44 , 1945.73 ) | 528 ( 496 , 560 ) | 691.92 ( 653.62 , 733.82 ) |
|  |  | Spain | 1500115 ( 1309401 , 1943247 ) | 1577.78 ( 1371.68 , 2040.56 ) | 641140 ( 619007 , 663963 ) | 650.09 ( 625.64 , 675.13 ) |
|  |  | Sweden | 467291 ( 408405 , 552455 ) | 2258.14 ( 1992.63 , 2611.24 ) | 180254 ( 175413 , 185018 ) | 821.23 ( 795.05 , 845.97 ) |
|  |  | Switzerland | 269060 ( 243197 , 331676 ) | 1513.92 ( 1370.32 , 1839.67 ) | 79895 ( 75431 , 84445 ) | 465.35 ( 440.01 , 493.19 ) |
|  |  | United Kingdom | 3218613 ( 2768100 , 3718940 ) | 2530.11 ( 2207.93 , 2947.33 ) | 736089 ( 659644 , 804849 ) | 582.89 ( 528.52 , 637.98 ) |
|  |  |  |  |  |  |  |

**Supplementary Table 3 Mortality of Ischemic heart disease and Ischemic stroke by country in Europe, 2023**

| **Location** | | | **Ischemic heart disease** | | **Ischemic stroke** | |
| --- | --- | --- | --- | --- | --- | --- |
|  |  |  | **Mortality number(95%UI)** | **ASMR (95%UI) per 100,000** | **Mortality number(95%UI)** | **ASMR (95%UI) per 100,000** |
|  | **Central Europe** |  | 307238 ( 284979 , 321075 ) | 131.63 ( 121.97 , 137.63 ) | 134966 ( 122833 , 143402 ) | 55.98 ( 50.86 , 59.5 ) |
|  |  | Albania | 5070 ( 4050 , 5992 ) | 119.26 ( 94.42 , 142.16 ) | 1038 ( 716 , 1433 ) | 24.45 ( 16.76 , 34.01 ) |
|  |  | Bosnia and Herzegovina | 8873 ( 7852 , 9990 ) | 141.74 ( 125.76 , 159.74 ) | 5472 ( 4759 , 6272 ) | 85.58 ( 74.07 , 98.15 ) |
|  |  | Bulgaria | 23483 ( 21840 , 24989 ) | 161.22 ( 149.41 , 171.95 ) | 17541 ( 15939 , 18551 ) | 115.45 ( 104.21 , 122.73 ) |
|  |  | Croatia | 10800 ( 9936 , 11455 ) | 115.5 ( 106.11 , 122.24 ) | 3939 ( 3544 , 4235 ) | 40.05 ( 35.84 , 43.19 ) |
|  |  | Czechia | 28948 ( 26556 , 30371 ) | 127.34 ( 116.59 , 133.76 ) | 6805 ( 6056 , 7334 ) | 29.1 ( 25.7 , 31.46 ) |
|  |  | Hungary | 34165 ( 31377 , 35701 ) | 165.37 ( 152.25 , 172.76 ) | 9131 ( 8394 , 9605 ) | 43.06 ( 39.6 , 45.35 ) |
|  |  | Montenegro | 1505 ( 1298 , 1714 ) | 139.99 ( 119.89 , 160.02 ) | 890 ( 744 , 1072 ) | 81.72 ( 68.25 , 98.37 ) |
|  |  | North Macedonia | 3959 ( 3526 , 4403 ) | 132.27 ( 117.36 , 147.68 ) | 3992 ( 3523 , 4457 ) | 130.71 ( 114.26 , 146.39 ) |
|  |  | Poland | 85411 ( 76596 , 90903 ) | 106.97 ( 96.52 , 113.67 ) | 29870 ( 25926 , 32855 ) | 36.65 ( 31.86 , 40.35 ) |
|  |  | Romania | 56445 ( 52456 , 59619 ) | 147.38 ( 137.02 , 155.72 ) | 31435 ( 28351 , 33962 ) | 78.94 ( 71.05 , 85.43 ) |
|  |  | Serbia | 26231 ( 22993 , 29739 ) | 181.12 ( 157.35 , 203.15 ) | 17803 ( 15516 , 20340 ) | 121.85 ( 107.44 , 138.72 ) |
|  |  | Slovakia | 15438 ( 13891 , 16627 ) | 161.25 ( 145.48 , 173.65 ) | 3641 ( 3227 , 4175 ) | 37.19 ( 32.84 , 42.79 ) |
|  |  | Slovenia | 2313 ( 2062 , 2526 ) | 44.1 ( 39.62 , 47.96 ) | 1391 ( 1193 , 1531 ) | 24.8 ( 21.36 , 27.24 ) |
|  | **Eastern Europe** |  | 831856 ( 778493 , 871407 ) | 230.46 ( 215.45 , 242.06 ) | 289938 ( 263302 , 309774 ) | 80.08 ( 72.3 , 85.61 ) |
|  |  | Belarus | 56956 ( 53333 , 60491 ) | 339.72 ( 317.58 , 360.83 ) | 11620 ( 10655 , 12497 ) | 68.27 ( 62.57 , 73.63 ) |
|  |  | Estonia | 2752 ( 2476 , 2941 ) | 83.73 ( 76.14 , 89.18 ) | 859 ( 762 , 933 ) | 25.38 ( 22.81 , 27.5 ) |
|  |  | Latvia | 6890 ( 6333 , 7321 ) | 150.29 ( 139.61 , 159.41 ) | 4278 ( 3877 , 4592 ) | 86.94 ( 79.34 , 92.8 ) |
|  |  | Lithuania | 12912 ( 11758 , 13600 ) | 193.03 ( 177.06 , 203.14 ) | 3500 ( 3148 , 3771 ) | 50.82 ( 45.84 , 54.57 ) |
|  |  | Republic of Moldova | 13055 ( 11827 , 13952 ) | 202.01 ( 182.9 , 215.74 ) | 2788 ( 2473 , 3118 ) | 42.86 ( 38.17 , 47.73 ) |
|  |  | Russian Federation | 498296 ( 465601 , 523049 ) | 203.96 ( 189.47 , 214.6 ) | 217956 ( 197484 , 234228 ) | 89.74 ( 80.64 , 96.71 ) |
|  |  | Ukraine | 240996 ( 219020 , 264289 ) | 306.85 ( 280.14 , 333.76 ) | 48936 ( 44019 , 54682 ) | 61.61 ( 55.65 , 68.88 ) |
|  | **Western Europe** |  | 564909 ( 484475 , 609429 ) | 48.91 ( 42.93 , 52.32 ) | 216173 ( 178666 , 238486 ) | 16.72 ( 13.99 , 18.36 ) |
|  |  | Andorra | 75 ( 56 , 97 ) | 35.22 ( 27.31 , 44.78 ) | 27 ( 19 , 37 ) | 11.08 ( 8.21 , 14.69 ) |
|  |  | Austria | 15951 ( 13864 , 17064 ) | 71.75 ( 63.14 , 76.54 ) | 3320 ( 2796 , 3663 ) | 14.06 ( 11.93 , 15.45 ) |
|  |  | Belgium | 10868 ( 9262 , 11832 ) | 38.27 ( 33.41 , 41.46 ) | 4640 ( 3836 , 5242 ) | 14.74 ( 12.36 , 16.56 ) |
|  |  | Cyprus | 1292 ( 1110 , 1518 ) | 69 ( 59.41 , 81.14 ) | 449 ( 362 , 562 ) | 24.49 ( 19.73 , 30.46 ) |
|  |  | Denmark | 5546 ( 4905 , 6062 ) | 40.37 ( 36.09 , 43.96 ) | 2709 ( 2288 , 2994 ) | 18.3 ( 15.47 , 20.2 ) |
|  |  | Finland | 12103 ( 10299 , 13157 ) | 77.74 ( 67.18 , 83.98 ) | 3523 ( 2911 , 3944 ) | 21 ( 17.54 , 23.44 ) |
|  |  | France | 54527 ( 46967 , 60012 ) | 30.63 ( 26.98 , 33.43 ) | 26349 ( 21736 , 29350 ) | 13.03 ( 10.91 , 14.45 ) |
|  |  | Germany | 167778 ( 141862 , 182064 ) | 69.78 ( 60.61 , 75.26 ) | 47973 ( 39358 , 53525 ) | 18.59 ( 15.49 , 20.55 ) |
|  |  | Greece | 20850 ( 18126 , 22669 ) | 70.24 ( 62.48 , 75.76 ) | 10106 ( 8342 , 11200 ) | 28.07 ( 23.3 , 30.94 ) |
|  |  | Iceland | 435 ( 379 , 474 ) | 65.59 ( 57.36 , 71.17 ) | 100 ( 83 , 113 ) | 14.35 ( 11.93 , 16.15 ) |
|  |  | Ireland | 5483 ( 4656 , 6068 ) | 60.28 ( 51.55 , 66.51 ) | 1264 ( 1029 , 1461 ) | 13.46 ( 10.95 , 15.56 ) |
|  |  | Israel | 4960 ( 4192 , 5467 ) | 34.08 ( 29.19 , 37.4 ) | 1583 ( 1284 , 1826 ) | 10.24 ( 8.43 , 11.78 ) |
|  |  | Italy | 78463 ( 64778 , 86823 ) | 41.49 ( 35.08 , 45.48 ) | 39299 ( 31055 , 44658 ) | 18.42 ( 14.75 , 20.85 ) |
|  |  | Luxembourg | 477 ( 428 , 514 ) | 41 ( 36.95 , 44.29 ) | 210 ( 180 , 232 ) | 17.33 ( 14.9 , 19.14 ) |
|  |  | Malta | 769 ( 665 , 844 ) | 70.22 ( 61.18 , 76.88 ) | 171 ( 141 , 193 ) | 14.95 ( 12.34 , 16.87 ) |
|  |  | Monaco | 34 ( 11 , 61 ) | 28.02 ( 8.43 , 50.91 ) | 24 ( 9 , 47 ) | 18.32 ( 6.61 , 36.47 ) |
|  |  | Netherlands | 14314 ( 12467 , 15616 ) | 34.57 ( 30.31 , 37.57 ) | 8160 ( 6880 , 8967 ) | 18.59 ( 15.73 , 20.43 ) |
|  |  | Norway | 4963 ( 4216 , 5428 ) | 41.74 ( 35.87 , 45.46 ) | 1755 ( 1424 , 1973 ) | 13.98 ( 11.42 , 15.68 ) |
|  |  | Portugal | 10530 ( 9109 , 11551 ) | 35.65 ( 31.66 , 39.02 ) | 9444 ( 7790 , 10503 ) | 27.03 ( 22.51 , 30.04 ) |
|  |  | San Marino | 25 ( 19 , 32 ) | 25.56 ( 19.36 , 32.89 ) | 18 ( 13 , 25 ) | 17.2 ( 12.64 , 23.46 ) |
|  |  | Spain | 41983 ( 35859 , 45653 ) | 33.79 ( 29.66 , 36.4 ) | 19531 ( 15788 , 22002 ) | 13.31 ( 10.97 , 14.88 ) |
|  |  | Sweden | 14127 ( 12150 , 15347 ) | 52.91 ( 46.27 , 57.01 ) | 4456 ( 3629 , 4939 ) | 15.18 ( 12.46 , 16.79 ) |
|  |  | Switzerland | 9678 ( 8032 , 10591 ) | 41.31 ( 35.15 , 44.97 ) | 3051 ( 2419 , 3434 ) | 12.12 ( 9.71 , 13.6 ) |
|  |  | United Kingdom | 89184 ( 80346 , 94712 ) | 58.86 ( 53.8 , 62.21 ) | 27821 ( 24119 , 30784 ) | 16.02 ( 14.04 , 17.71 ) |
|  |  |  |  |  |  |  |

**Supplementary Table 4. DALYs of ischemic heart disease and ischemic stroke by country in Europe, 2023**

| **Location** | | | **Ischemic heart disease** | | **Ischemic stroke** | |
| --- | --- | --- | --- | --- | --- | --- |
|  |  |  | **DALYS number(95%UI)** | **ASDR (95%UI) per 100,000** | **DALYS number(95%UI)** | **ASDR (95%UI) per 100,000** |
|  | **Central Europe** |  | 5236744 ( 4940999 , 5425850 ) | 2340.46 ( 2216.32 , 2423.48 ) | 2237453 ( 2067612 , 2377885 ) | 945.58 ( 871.76 , 1007.11 ) |
|  |  | Albania | 88870 ( 73516 , 103381 ) | 2075.29 ( 1713.26 , 2423.74 ) | 18816 ( 14046 , 24247 ) | 432.23 ( 325.31 , 555.33 ) |
|  |  | Bosnia and Herzegovina | 158347 ( 144107 , 175521 ) | 2589.14 ( 2358.75 , 2878.27 ) | 91767 ( 81004 , 103589 ) | 1437 ( 1266.31 , 1621.71 ) |
|  |  | Bulgaria | 439114 ( 414937 , 464738 ) | 3151.27 ( 2982.88 , 3352.54 ) | 293644 ( 270513 , 312300 ) | 1901.34 ( 1743.36 , 2024.9 ) |
|  |  | Czechia | 460832 ( 430814 , 480232 ) | 2109.68 ( 1969.08 , 2199.95 ) | 116683 ( 105422 , 126607 ) | 512.56 ( 461.84 , 557.52 ) |
|  |  | Hungary | 581487 ( 546085 , 604779 ) | 2986.4 ( 2810.45 , 3105.32 ) | 164965 ( 153107 , 175545 ) | 816.96 ( 758.94 , 871.58 ) |
|  |  | Montenegro | 27852 ( 24553 , 31475 ) | 2579.18 ( 2259.67 , 2917.16 ) | 14202 ( 12054 , 17007 ) | 1264.56 ( 1072.37 , 1517.56 ) |
|  |  | North Macedonia | 71100 ( 64434 , 79548 ) | 2288.58 ( 2065.58 , 2555.92 ) | 64126 ( 57734 , 71435 ) | 1971.31 ( 1772.46 , 2192.77 ) |
|  |  | Poland | 1429949 ( 1329725 , 1506110 ) | 1900.79 ( 1770.55 , 2000.76 ) | 519423 ( 465186 , 568191 ) | 668.4 ( 599.74 , 729.63 ) |
|  |  | Romania | 987679 ( 922555 , 1035689 ) | 2704.26 ( 2522.43 , 2838.09 ) | 489133 ( 445497 , 528732 ) | 1236.6 ( 1128.27 , 1339.67 ) |
|  |  | Serbia | 442854 ( 392458 , 493602 ) | 2886.82 ( 2572.21 , 3199.39 ) | 279764 ( 246742 , 314921 ) | 1778.24 ( 1582.67 , 1998.8 ) |
|  |  | Slovakia | 263153 ( 239283 , 282376 ) | 2742.4 ( 2487.66 , 2937.42 ) | 69151 ( 61505 , 78401 ) | 699.33 ( 619.68 , 788.56 ) |
|  |  | Slovenia | 37975 ( 34968 , 40810 ) | 809.99 ( 749.46 , 871.58 ) | 20678 ( 18172 , 22565 ) | 399.86 ( 354.36 , 435.16 ) |
|  | **Eastern Europe** |  | 16039890 ( 15301312 , 16661721 ) | 4457.25 ( 4244.5 , 4629.92 ) | 5162301 ( 4820701 , 5571946 ) | 1406.23 ( 1310.98 , 1518.5 ) |
|  |  | Belarus | 1049502 ( 995757 , 1110689 ) | 6359.38 ( 6033.83 , 6708.36 ) | 211913 ( 195485 , 227962 ) | 1249.55 ( 1154.77 , 1347.2 ) |
|  |  | Estonia | 44462 ( 40931 , 46928 ) | 1530.64 ( 1421.5 , 1616.33 ) | 14770 ( 13383 , 16036 ) | 491.77 ( 446.43 , 538.69 ) |
|  |  | Latvia | 115519 ( 108324 , 122016 ) | 2849.25 ( 2681.17 , 3020.43 ) | 63783 ( 58653 , 68051 ) | 1414.35 ( 1298.6 , 1514.24 ) |
|  |  | Lithuania | 205629 ( 191062 , 214875 ) | 3342.98 ( 3134.98 , 3485.57 ) | 57500 ( 53035 , 61758 ) | 908.61 ( 842.24 , 978.67 ) |
|  |  | Republic of Moldova | 250298 ( 230861 , 263810 ) | 4005.76 ( 3721.46 , 4213.27 ) | 56389 ( 50605 , 62608 ) | 884.44 ( 794.46 , 979.44 ) |
|  |  | Russian Federation | 9909579 ( 9467201 , 10302616 ) | 4018.8 ( 3829.44 , 4183.66 ) | 3836490 ( 3551174 , 4127034 ) | 1532.63 ( 1415.64 , 1651.85 ) |
|  |  | Ukraine | 4464901 ( 4135795 , 4814012 ) | 5783.16 ( 5367.68 , 6217.26 ) | 921456 ( 829879 , 1019602 ) | 1171.87 ( 1055.73 , 1293.5 ) |
|  | **Western Europe** |  | 9272217 ( 8353447 , 9898738 ) | 949.57 ( 869.37 , 1009.07 ) | 3427927 ( 2939219 , 3766797 ) | 306.92 ( 269.52 , 340.6 ) |
|  |  | Andorra | 1292 ( 1026 , 1608 ) | 730.11 ( 591.36 , 904.86 ) | 432 ( 342 , 549 ) | 220.74 ( 177.33 , 275.57 ) |
|  |  | Austria | 250217 ( 226284 , 266671 ) | 1266.1 ( 1156.08 , 1346.09 ) | 59520 ( 51599 , 66329 ) | 284.64 ( 248 , 320.22 ) |
|  |  | Belgium | 178242 ( 157983 , 191010 ) | 732.02 ( 662.33 , 781.65 ) | 71697 ( 60606 , 79757 ) | 261.8 ( 224.69 , 292.11 ) |
|  |  | Cyprus | 22592 ( 19569 , 25948 ) | 1186.26 ( 1026.22 , 1357.95 ) | 6557 ( 5397 , 7772 ) | 346.2 ( 285.24 , 413.07 ) |
|  |  | Denmark | 95671 ( 86915 , 102697 ) | 784.9 ( 720.09 , 839.99 ) | 42793 ( 37145 , 46915 ) | 312.71 ( 271.37 , 343.89 ) |
|  |  | Finland | 184668 ( 165418 , 198289 ) | 1368.3 ( 1238.68 , 1465.67 ) | 54073 ( 46083 , 59538 ) | 363.9 ( 315.25 , 403.53 ) |
|  |  | France | 934053 ( 835236 , 1008604 ) | 635.94 ( 579.91 , 686.36 ) | 426262 ( 368761 , 471106 ) | 251.12 ( 220.59 , 279.63 ) |
|  |  | Germany | 2661178 ( 2382772 , 2841584 ) | 1306.83 ( 1188.75 , 1391.9 ) | 877053 ( 771851 , 973021 ) | 397.49 ( 351.69 , 442.71 ) |
|  |  | Greece | 347806 ( 316344 , 368545 ) | 1439.79 ( 1350.9 , 1521.33 ) | 133294 ( 112191 , 146219 ) | 418.99 ( 358.27 , 460.77 ) |
|  |  | Iceland | 7054 ( 6384 , 7594 ) | 1148.19 ( 1041.15 , 1238.26 ) | 1586 ( 1378 , 1776 ) | 244 ( 211.97 , 273.98 ) |
|  |  | Ireland | 92014 ( 81384 , 100476 ) | 1066.23 ( 942.87 , 1163.98 ) | 19776 ( 16576 , 22283 ) | 220.06 ( 184.5 , 248.03 ) |
|  |  | Israel | 83016 ( 74501 , 89845 ) | 629.95 ( 571.38 , 679.88 ) | 29107 ( 24815 , 32830 ) | 209.63 ( 179.44 , 237.97 ) |
|  |  | Italy | 1191198 ( 1029847 , 1306915 ) | 772.05 ( 684.66 , 842.37 ) | 530719 ( 441422 , 596797 ) | 288.18 ( 240.75 , 325.52 ) |
|  |  | Luxembourg | 7826 ( 7133 , 8424 ) | 712.05 ( 653.91 , 764.48 ) | 3092 ( 2744 , 3369 ) | 269.97 ( 242.55 , 294.96 ) |
|  |  | Malta | 12785 ( 11552 , 13780 ) | 1251.32 ( 1131.7 , 1340.69 ) | 2729 ( 2336 , 3033 ) | 248.01 ( 213.79 , 275.7 ) |
|  |  | Monaco | 530 ( 179 , 923 ) | 505.57 ( 163.8 , 874.6 ) | 358 ( 172 , 626 ) | 306.49 ( 154.15 , 521.92 ) |
|  |  | Netherlands | 237202 ( 214226 , 254980 ) | 636.54 ( 578.8 , 681.85 ) | 128209 ( 110711 , 140864 ) | 317.71 ( 276.27 , 349.48 ) |
|  |  | Norway | 85235 ( 74651 , 93459 ) | 802.46 ( 709.22 , 876.47 ) | 31106 ( 26296 , 35149 ) | 274.33 ( 235.61 , 312.44 ) |
|  |  | Portugal | 184767 ( 166310 , 200085 ) | 762.81 ( 693.65 , 824.01 ) | 132683 ( 113224 , 146014 ) | 434.25 ( 376.83 , 480.48 ) |
|  |  | San Marino | 412 ( 330 , 503 ) | 486.33 ( 389.75 , 592.07 ) | 287 ( 228 , 359 ) | 308.22 ( 248.54 , 384.13 ) |
|  |  | Spain | 717274 ( 645226 , 771462 ) | 698.58 ( 640.86 , 743.42 ) | 315538 ( 269235 , 353268 ) | 258.96 ( 225.58 , 292.24 ) |
|  |  | Sweden | 220346 ( 198209 , 234709 ) | 956.92 ( 871.8 , 1014.97 ) | 78508 ( 67732 , 87032 ) | 302.78 ( 263.31 , 338.97 ) |
|  |  | Switzerland | 139209 ( 122051 , 150445 ) | 688.93 ( 615.09 , 739.88 ) | 45468 ( 38173 , 50947 ) | 208.2 ( 175.88 , 234.6 ) |
|  |  | United Kingdom | 1609534 ( 1491587 , 1693204 ) | 1221.59 ( 1142.34 , 1281.33 ) | 434086 ( 382513 , 476928 ) | 282.11 ( 250.66 , 311.9 ) |
|  |  |  |  |  |  |  |

**Supplementary Table 5 ASPR for Joinpoint**

| **location** | **IHD** | | | | | | | **IS** | | | | | | |
| --- | --- | --- | --- | --- | --- | --- | --- | --- | --- | --- | --- | --- | --- | --- |
|  | **Range** | **APC（%）** | **Lower CI（%）** | **Upper CI（%）** | **t** | **P** | **AAPC（%）** | **Range** | **APC（%）** | **Lower CI（%）** | **Upper CI（%）** | **t** | **P** | **AAPC（%）** |
| Central Europe | 1990-1996 | -0.14 | -0.49 | 0.22 | -0.8 | 0.434 | -1.56* (-1.81--1.32) | 1990-1996 | -0.09 | -0.25 | 0.07 | -1.25 | 0.23 | -1.05* (-1.15--0.95) |
|  | 1996-2001 | -2.53* | -3.24 | -1.81 | -7.34 | < 0.001 |  | 1996-1999 | -1.31* | -2.18 | -0.44 | -3.15 | 0.006 |  |
|  | 2001-2005 | -4.44* | -5.63 | -3.24 | -7.67 | < 0.001 |  | 1999-2006 | -0.64* | -0.78 | -0.49 | -9.15 | < 0.001 |  |
|  | 2005-2009 | 1.16* | 0.04 | 2.28 | 2.18 | 0.043 |  | 2006-2014 | -2.25* | -2.36 | -2.13 | -41.32 | < 0.001 |  |
|  | 2009-2020 | -1.28* | -1.44 | -1.12 | -16.6 | < 0.001 |  | 2014-2020 | -0.27* | -0.45 | -0.1 | -3.34 | 0.004 |  |
|  | 2020-2023 | -3.48* | -4.57 | -2.38 | -6.61 | < 0.001 |  | 2020-2023 | -1.99* | -2.37 | -1.62 | -11.05 | < 0.001 |  |
| Eastern Europe | 1990-2000 | 0.58* | 0.3 | 0.87 | 4.2 | < 0.001 | -0.47* (-0.61--0.33) | 1990-1995 | 0.25* | 0.09 | 0.41 | 3.38 | 0.004 | -0.72* (-0.78--0.66) |
|  | 2000-2013 | -1.22* | -1.43 | -1 | -11.44 | < 0.001 |  | 1995-2000 | -0.41* | -0.61 | -0.21 | -4.25 | 0.001 |  |
|  | 2013-2023 | -0.54* | -0.84 | -0.24 | -3.73 | 0.001 |  | 2000-2005 | 0.18 | -0.03 | 0.38 | 1.84 | 0.084 |  |
|  |  |  |  |  |  |  |  | 2005-2014 | -2.06* | -2.13 | -1.99 | -62.16 | < 0.001 |  |
|  |  |  |  |  |  |  |  | 2014-2020 | -0.19* | -0.32 | -0.06 | -3.07 | 0.007 |  |
|  |  |  |  |  |  |  |  | 2020-2023 | -1.31* | -1.6 | -1.03 | -9.58 | < 0.001 |  |
| Western Europe | 1990-1996 | -0.67* | -0.82 | -0.52 | -9.4 | < 0.001 | -1.76* (-1.86--1.66) | 1990-1996 | -0.08 | -0.23 | 0.08 | -1.06 | 0.306 | -0.75* (-0.84--0.66) |
|  | 1996-2001 | -2.21* | -2.45 | -1.98 | -19.83 | < 0.001 |  | 1996-1999 | -1.29* | -2.1 | -0.48 | -3.34 | 0.004 |  |
|  | 2001-2004 | -3.75* | -4.51 | -2.98 | -10.15 | < 0.001 |  | 1999-2010 | -0.72* | -0.77 | -0.66 | -27.11 | < 0.001 |  |
|  | 2004-2015 | -2.35* | -2.4 | -2.29 | -88.87 | < 0.001 |  | 2010-2014 | -1.92* | -2.22 | -1.62 | -13.33 | < 0.001 |  |
|  | 2015-2020 | -1.62* | -1.88 | -1.36 | -13.01 | < 0.001 |  | 2014-2020 | -0.15* | -0.27 | -0.02 | -2.49 | 0.023 |  |
|  | 2020-2023 | 0.77* | 0.22 | 1.32 | 2.94 | 0.009 |  | 2020-2023 | -1.26* | -1.57 | -0.95 | -8.59 | < 0.001 |  |

**Supplementary Table 6 ASIR for Joinpoint**

| **location** | **IHD** | | | | | | | **IS** | | | | | | |
| --- | --- | --- | --- | --- | --- | --- | --- | --- | --- | --- | --- | --- | --- | --- |
|  | **Range** | **APC（%）** | **Lower CI（%）** | **Upper CI（%）** | **t** | **P** | **AAPC（%）** | **Range** | **APC（%）** | **Lower CI（%）** | **Upper CI（%）** | **t** | **P** | **AAPC（%）** |
| Central Europe | 1990-1993 | -0.55* | -0.85 | -0.26 | -3.93 | 0.001 | -2.51* (-2.59--2.44) | 1990-1995 | -0.82* | -0.94 | -0.7 | -14.26 | < 0.001 | -1.94* (-1.99--1.9) |
|  | 1993-1996 | -1.24* | -1.81 | -0.66 | -4.5 | < 0.001 |  | 1995-2000 | -2.49* | -2.64 | -2.33 | -34.14 | < 0.001 |  |
|  | 1996-2011 | -2.81* | -2.83 | -2.78 | -211.68 | < 0.001 |  | 2000-2010 | -1.76* | -1.8 | -1.72 | -88.38 | < 0.001 |  |
|  | 2011-2014 | -4.86* | -5.39 | -4.32 | -18.66 | < 0.001 |  | 2010-2015 | -4.51* | -4.64 | -4.38 | -72.07 | < 0.001 |  |
|  | 2014-2019 | -3.01* | -3.18 | -2.84 | -37.66 | < 0.001 |  | 2015-2019 | -1.42* | -1.64 | -1.21 | -13.8 | < 0.001 |  |
|  | 2019-2023 | -1.42* | -1.62 | -1.21 | -14.63 | < 0.001 |  | 2019-2023 | -0.37* | -0.52 | -0.23 | -5.49 | < 0.001 |  |
| Eastern Europe | 1990-2000 | 1.88* | 1.8 | 1.96 | 49.74 | < 0.001 | -1.83* (-1.96--1.7) | 1990-1995 | -0.68* | -0.99 | -0.36 | -4.49 | < 0.001 | -1.38* (-1.56--1.2) |
|  | 2000-2005 | -0.90* | -1.25 | -0.56 | -5.42 | < 0.001 |  | 1995-2000 | 0.85* | 0.42 | 1.29 | 4.14 | 0.001 |  |
|  | 2005-2008 | -3.45* | -4.62 | -2.27 | -6.02 | < 0.001 |  | 2000-2011 | -2.22* | -2.32 | -2.11 | -43.03 | < 0.001 |  |
|  | 2008-2019 | -5.16* | -5.26 | -5.06 | -105.55 | < 0.001 |  | 2011-2014 | -5.28* | -6.53 | -4.02 | -8.66 | < 0.001 |  |
|  | 2019-2023 | -1.53* | -1.97 | -1.08 | -7.14 | < 0.001 |  | 2014-2017 | -1.67* | -2.9 | -0.44 | -2.84 | 0.011 |  |
|  |  |  |  |  |  |  |  | 2017-2023 | -0.13 | -0.35 | 0.1 | -1.19 | 0.252 |  |
| Western Europe | 1990-2001 | 0.45* | 0.18 | 0.71 | 3.52 | 0.002 | -1.48* (-1.68--1.29) | 1990-1995 | -1.62* | -1.79 | -1.44 | -19.49 | < 0.001 | -1.92* (-1.99--1.85) |
|  | 2001-2010 | -1.00* | -1.32 | -0.68 | -6.4 | < 0.001 |  | 1995-2003 | -2.62* | -2.71 | -2.53 | -60.5 | < 0.001 |  |
|  | 2010-2017 | -4.88* | -5.39 | -4.38 | -19.5 | < 0.001 |  | 2003-2011 | -1.74* | -1.81 | -1.66 | -46.52 | < 0.001 |  |
|  | 2017-2023 | -1.68* | -2.38 | -0.98 | -4.94 | < 0.001 |  | 2011-2014 | -2.83* | -3.37 | -2.29 | -10.94 | < 0.001 |  |
|  |  |  |  |  |  |  |  | 2014-2020 | -1.07* | -1.2 | -0.93 | -16.54 | < 0.001 |  |
|  |  |  |  |  |  |  |  | 2020-2023 | -1.87* | -2.25 | -1.48 | -10.2 | < 0.001 |  |

**Supplementary Table 7 ASMR for Joinpoint**

| **location** | **IHD** | | | | | | | **IS** | | | | | | |
| --- | --- | --- | --- | --- | --- | --- | --- | --- | --- | --- | --- | --- | --- | --- |
|  | **Range** | **APC（%）** | **Lower CI（%）** | **Upper CI（%）** | **t** | **P** | **AAPC（%）** | **Range** | **APC（%）** | **Lower CI（%）** | **Upper CI（%）** | **t** | **P** | **AAPC（%）** |
| Central Europe | 1990-1997 | -1.01* | -1.27 | -0.75 | -8.08 | < 0.001 | -2.18* (-2.36--2) | 1990-1996 | -0.73* | -1 | -0.46 | -5.76 | < 0.001 | -2.83* (-3.05--2.62) |
|  | 1997-2001 | -3.99* | -4.91 | -3.06 | -8.78 | < 0.001 |  | 1996-2000 | -3.09* | -3.89 | -2.29 | -8.02 | < 0.001 |  |
|  | 2001-2009 | -2.24* | -2.52 | -1.96 | -16.57 | < 0.001 |  | 2000-2003 | -1.97* | -3.59 | -0.33 | -2.53 | 0.021 |  |
|  | 2009-2014 | -3.13* | -3.85 | -2.41 | -8.91 | < 0.001 |  | 2003-2014 | -3.83* | -3.96 | -3.7 | -59.56 | < 0.001 |  |
|  | 2014-2023 | -1.70* | -1.93 | -1.47 | -15.4 | < 0.001 |  | 2014-2021 | -2.66* | -2.97 | -2.35 | -18.01 | < 0.001 |  |
|  |  |  |  |  |  |  |  | 2021-2023 | -4.91* | -6.93 | -2.86 | -4.96 | < 0.001 |  |
| Eastern Europe | 1990-1994 | 5.84* | 4.24 | 7.48 | 7.74 | < 0.001 | -1.11* (-1.53--0.7) | 1990-1994 | 5.19* | 3.32 | 7.1 | 5.87 | < 0.001 | -2.18* (-2.65--1.7) |
|  | 1994-1998 | -2.07 | -4.25 | 0.16 | -1.94 | 0.067 |  | 1994-1998 | -2.46 | -5.02 | 0.17 | -1.96 | 0.065 |  |
|  | 1998-2003 | 1.78* | 0.29 | 3.29 | 2.49 | 0.022 |  | 1998-2003 | -0.17 | -1.85 | 1.54 | -0.21 | 0.838 |  |
|  | 2003-2010 | -2.24* | -3.01 | -1.48 | -6.06 | < 0.001 |  | 2003-2013 | -5.11* | -5.6 | -4.62 | -21.11 | < 0.001 |  |
|  | 2010-2023 | -3.34* | -3.62 | -3.07 | -24.77 | < 0.001 |  | 2013-2023 | -2.92* | -3.41 | -2.42 | -12.11 | < 0.001 |  |
| Western Europe | 1990-1998 | -2.87* | -3.12 | -2.62 | -23.49 | < 0.001 | -3.26* (-3.46--3.06) | 1990-2003 | -4.27* | -4.41 | -4.13 | -61.57 | < 0.001 | -3.85* (-4.22--3.48) |
|  | 1998-2013 | -4.31* | -4.45 | -4.17 | -63.15 | < 0.001 |  | 2003-2006 | -6.10* | -9.01 | -3.09 | -4.16 | < 0.001 |  |
|  | 2013-2019 | -3.11* | -3.87 | -2.35 | -8.3 | < 0.001 |  | 2006-2013 | -4.54* | -5.09 | -3.98 | -16.68 | < 0.001 |  |
|  | 2019-2023 | -0.28 | -1.38 | 0.83 | -0.52 | 0.606 |  | 2013-2021 | -2.89* | -3.37 | -2.41 | -12.29 | < 0.001 |  |
|  |  |  |  |  |  |  |  | 2021-2023 | 0.97 | -2.81 | 4.9 | 0.53 | 0.603 |  |
|  |  |  |  |  |  |  |  |  |  |  |  |  |  |  |

**Supplementary Table 8 ASDR for Joinpoint**

| **location** | **IHD** | | | | | | | **IS** | | | | | | |
| --- | --- | --- | --- | --- | --- | --- | --- | --- | --- | --- | --- | --- | --- | --- |
|  | **Range** | **APC（%）** | **Lower CI（%）** | **Upper CI（%）** | **t** | **P** | **AAPC（%）** | **Range** | **APC（%）** | **Lower CI（%）** | **Upper CI（%）** | **t** | **P** | **AAPC（%）** |
| Central Europe | 1990-1992 | -0.06 | -1.68 | 1.58 | -0.08 | 0.936 | -2.38* (-2.56--2.2) | 1990-1996 | -0.77* | -0.99 | -0.54 | -7.14 | < 0.001 | -2.73* (-2.89--2.56) |
|  | 1992-1997 | -1.48* | -2 | -0.96 | -5.97 | < 0.001 |  | 1996-2000 | -2.93* | -3.6 | -2.26 | -9.1 | < 0.001 |  |
|  | 1997-2001 | -4.34* | -5.16 | -3.51 | -10.87 | < 0.001 |  | 2000-2004 | -2.23* | -2.9 | -1.56 | -6.95 | < 0.001 |  |
|  | 2001-2009 | -2.66* | -2.89 | -2.42 | -23.76 | < 0.001 |  | 2004-2014 | -3.93* | -4.06 | -3.8 | -61.15 | < 0.001 |  |
|  | 2009-2014 | -3.38* | -3.95 | -2.8 | -12.2 | < 0.001 |  | 2014-2021 | -2.31* | -2.57 | -2.05 | -18.27 | < 0.001 |  |
|  | 2014-2023 | -1.70* | -1.88 | -1.51 | -19.33 | < 0.001 |  | 2021-2023 | -4.49* | -6.16 | -2.8 | -5.51 | < 0.001 |  |
| Eastern Europe | 1990-1994 | 7.99* | 6.14 | 9.88 | 9.37 | < 0.001 | -1.01* (-1.48--0.55) | 1990-1994 | 6.07* | 4.19 | 7.97 | 6.9 | < 0.001 | -2.07* (-2.51--1.63) |
|  | 1994-1998 | -2.99* | -5.25 | -0.68 | -2.73 | 0.014 |  | 1994-1998 | -2.94* | -5.3 | -0.53 | -2.53 | 0.02 |  |
|  | 1998-2003 | 2.65* | 1.22 | 4.09 | 3.95 | 0.001 |  | 1998-2003 | 0.27 | -1.33 | 1.89 | 0.35 | 0.73 |  |
|  | 2003-2010 | -3.02* | -3.73 | -2.3 | -8.78 | < 0.001 |  | 2003-2013 | -5.15* | -5.59 | -4.71 | -23.81 | < 0.001 |  |
|  | 2010-2017 | -3.97* | -4.77 | -3.17 | -10.27 | < 0.001 |  | 2013-2023 | -2.87* | -3.31 | -2.42 | -13.19 | < 0.001 |  |
|  | 2017-2023 | -2.53* | -3.44 | -1.62 | -5.83 | < 0.001 |  |  |  |  |  |  |  |  |
| Western Europe | 1990-1998 | -3.21* | -3.43 | -2.99 | -29.36 | < 0.001 | -3.26* (-3.43--3.1) | 1990-2003 | -4.05* | -4.17 | -3.92 | -64.84 | < 0.001 | -3.45* (-3.73--3.17) |
|  | 1998-2012 | -4.47* | -4.6 | -4.34 | -70.13 | < 0.001 |  | 2003-2006 | -5.31* | -8.03 | -2.51 | -3.87 | 0.001 |  |
|  | 2012-2019 | -2.75* | -3.25 | -2.25 | -11.28 | < 0.001 |  | 2006-2014 | -3.89* | -4.31 | -3.47 | -18.85 | < 0.001 |  |
|  | 2019-2023 | 0.04 | -0.97 | 1.05 | 0.07 | 0.943 |  | 2014-2023 | -1.55* | -1.85 | -1.24 | -10.35 | < 0.001 |  |

**Supplementary Table 9. Age-specific prevalence of ischemic heart disease and ischemic stroke in Europe, 2023**

| **Location** | | **Ischemic heart disease** | | **Ischemic stroke** | |
| --- | --- | --- | --- | --- | --- |
|  |  | **Prevalence number(95%UI)** | **ASPR (95%UI) per 100,000** | **Prevalence number(95%UI)** | **ASPR (95%UI) per 100,000** |
| **Europe** | 0 to 4 | 0(0-0) | 0(0-0) | 4299.97(3675.17-4955.55) | 12.4(11.02-13.77) |
|  | 5 to 9 | 0(0-0) | 0(0-0) | 14166.21(11370.06-17368.25) | 35.21(29.43-41.53) |
|  | 10 to 14 | 0(0-0) | 0(0-0) | 24919.55(20074.85-30677.82) | 58.21(48.9-68.95) |
|  | 15 to 19 | 16648.62(9144.3-25878.2) | 40.06(22.91-59.97) | 34317.39(28387.91-41911.77) | 82.58(71.13-97.13) |
|  | 20 to 24 | 45454.38(28403.14-66997.62) | 110.27(71.68-156.66) | 45253.31(37885.96-54556.17) | 109.78(95.61-127.57) |
|  | 25 to 29 | 103000.04(74521.87-138468.46) | 239.04(179.9-309.68) | 62657.34(53089.9-73573.7) | 145.41(128.16-164.55) |
|  | 30 to 34 | 245963(185649.32-333023.71) | 488.96(384.85-636.92) | 100566.39(85776.71-117855.72) | 199.92(177.81-225.4) |
|  | 35 to 39 | 541497.06(430044.56-684806.82) | 971.4(806.34-1179.25) | 164501.72(142802.26-188030.13) | 295.1(267.76-323.79) |
|  | 40 to 44 | 965114.89(769919.39-1198222.54) | 1737.49(1445.93-2072.81) | 242020.32(209698.43-276303.4) | 435.71(393.82-477.98) |
|  | 45 to 49 | 1751062.16(1447842.1-2079863.07) | 3237.64(2788.68-3701.24) | 358245.48(313960.82-405617.64) | 662.38(604.72-721.82) |
|  | 50 to 54 | 2939189.99(2442271.28-3496690.19) | 5513.96(4765.02-6320.85) | 535008.73(472395-608470.25) | 1003.68(921.67-1099.91) |
|  | 55 to 59 | 4477141.6(3792701.75-5250642.23) | 8558.12(7534.36-9678.16) | 795858.88(725122.29-880748.95) | 1521.3(1440.49-1623.43) |
|  | 60 to 64 | 6428620.05(5412397.67-7555978.16) | 12509.3(10974.94-14142.98) | 1169713.76(1062432.87-1297728.07) | 2276.12(2154.34-2429.04) |
|  | 65 to 69 | 7206063(6129204.13-8488062.79) | 15659.84(13878.1-17739.09) | 1451952.17(1327636.92-1618218.28) | 3155.31(3006.11-3381.89) |
|  | 70 to 74 | 6998495.58(5973203.43-8333398.33) | 17942.17(15937.65-20572.47) | 1594910.1(1438699.48-1785462.31) | 4088.9(3838.73-4407.73) |
|  | 75 to 79 | 5392776.16(4529115.49-6403701.05) | 19638.07(17085.34-22538.05) | 1376999.06(1258668.58-1517969.56) | 5014.41(4748.12-5342.55) |
|  | 80 to 84 | 4796544.78(3886350.22-5726741.76) | 23461.13(19727.53-27033.19) | 1263209.48(1158734.47-1369692.38) | 6178.68(5881.86-6465.66) |
|  | 85 to 89 | 3260688.66(2573367.65-3909315.51) | 25588.19(20947.33-29610.2) | 921102.49(841149.65-996915.5) | 7228.33(6847-7550.9) |
|  | 90 to 94 | 1254488.57(980591.52-1550167.22) | 25361.04(20501.05-30345.85) | 405673.31(365808.45-443037.82) | 8201.19(7647.89-8672.85) |
|  | 95 plus | 326879.77(238403.89-447586.55) | 23503.63(17713.07-31176.68) | 126324.65(113722.79-139019.87) | 9083.12(8449.44-9683.44) |

**Supplementary Table 10. Age-specific incidence of ischemic heart disease and ischemic stroke in Europe, 2023**

| **Location** | | **Ischemic heart disease** | | **Ischemic stroke** | |
| --- | --- | --- | --- | --- | --- |
|  |  | **Incidence number(95%UI)** | **ASIR (95%UI) per 100,000** | **Incidence number(95%UI)** | **ASIR (95%UI) per 100,000** |
| **Europe** | 0 to 4 | 0(0-0) | 0(0-0) | 1585.19(728.51-2926.8) | 4.57(2.18-8.13) |
|  | 5 to 9 | 0(0-0) | 0(0-0) | 1754.67(532.56-3506.73) | 4.36(1.38-8.39) |
|  | 10 to 14 | 0(0-0) | 0(0-0) | 1889.5(510.32-3817.72) | 4.41(1.24-8.58) |
|  | 15 to 19 | 653.48(129.08-1740.7) | 1.57(0.32-4.03) | 1888.75(632.03-3852.73) | 4.54(1.58-8.93) |
|  | 20 to 24 | 1763.78(790.31-3421.16) | 4.28(1.99-8) | 2348.15(1003.56-4555.5) | 5.7(2.53-10.65) |
|  | 25 to 29 | 2878.08(1179.83-5732.56) | 6.68(2.85-12.82) | 3367.4(1347.64-6322.63) | 7.81(3.25-14.14) |
|  | 30 to 34 | 8280.53(5512.71-12737.22) | 16.46(11.43-24.36) | 6954.9(3970.79-10438.47) | 13.83(8.23-19.96) |
|  | 35 to 39 | 18961.53(11873.29-29402.09) | 34.02(22.26-50.63) | 13300.57(7989.47-20534.42) | 23.86(14.98-35.36) |
|  | 40 to 44 | 43177.45(31492.9-55979.74) | 77.73(59.14-96.84) | 22269.1(16163.41-29612.46) | 40.09(30.36-51.23) |
|  | 45 to 49 | 80951.77(54692.98-112253.23) | 149.68(105.34-199.76) | 33819.78(22966.43-45717.45) | 62.53(44.24-81.36) |
|  | 50 to 54 | 138053.03(102207.08-174244.42) | 258.99(199.41-314.98) | 50744.52(37892.11-64923.46) | 95.2(73.93-117.36) |
|  | 55 to 59 | 211240.24(146611.98-292537.14) | 403.79(291.25-539.21) | 72018.18(51748.64-96573.04) | 137.66(102.8-178.01) |
|  | 60 to 64 | 313161.65(240209.54-402175.66) | 609.37(487.08-752.78) | 109547.42(82350.71-141050.74) | 213.17(166.99-264.01) |
|  | 65 to 69 | 385868.51(267898.02-522764.18) | 838.55(606.59-1092.52) | 141548.83(97600.38-193293.4) | 307.61(220.99-403.96) |
|  | 70 to 74 | 429348.31(329453.32-553455.04) | 1100.73(879.04-1366.3) | 168668.87(129962.88-218857.34) | 432.42(346.77-540.29) |
|  | 75 to 79 | 368429.93(255227-513562.07) | 1341.66(962.8-1807.5) | 154127.12(115883.09-198336.46) | 561.26(437.15-698.05) |
|  | 80 to 84 | 399519.87(307909.69-515449.35) | 1954.15(1562.98-2433.19) | 191469.94(156029.94-230642.2) | 936.53(792.02-1088.75) |
|  | 85 to 89 | 344953.85(240715.38-470449.62) | 2707.02(1959.43-3563.31) | 189620.45(159665.35-221580.88) | 1488.04(1299.68-1678.31) |
|  | 90 to 94 | 170212.17(129518.22-218412.27) | 3441.05(2707.81-4275.61) | 93681.54(76809.59-110892.18) | 1893.89(1605.84-2170.81) |
|  | 95 plus | 63082.38(41835.65-90401.09) | 4535.81(3108.33-6296.9) | 32069.85(25745.12-38952.09) | 2305.92(1912.83-2713.21) |

**Supplementary Table 11. Age-specific mortality of ischemic heart disease and ischemic stroke in Europe, 2023**

| **Location** | | **Ischemic heart disease** | | **Ischemic stroke** | |
| --- | --- | --- | --- | --- | --- |
|  |  | **Mortality number(95%UI)** | **ASMR (95%UI) per 100,000** | **Mortality number(95%UI)** | **ASMR (95%UI) per 100,000** |
| **Europe** | 0 to 4 | 0(0-0) | 0(0-0) | 4.31(3.37-5.44) | 0.01(0.01-0.02) |
|  | 5 to 9 | 0(0-0) | 0(0-0) | 1.86(1.45-2.38) | 0(0-0.01) |
|  | 10 to 14 | 0(0-0) | 0(0-0) | 2.99(2.32-3.84) | 0.01(0.01-0.01) |
|  | 15 to 19 | 98.2(83.69-115.54) | 0.24(0.21-0.27) | 16.54(13.25-21.39) | 0.04(0.03-0.05) |
|  | 20 to 24 | 257.03(218.43-296.91) | 0.62(0.55-0.69) | 29.61(24.26-36.93) | 0.07(0.06-0.09) |
|  | 25 to 29 | 644.44(558.68-739.68) | 1.5(1.35-1.65) | 64.11(53.55-79.47) | 0.15(0.13-0.18) |
|  | 30 to 34 | 2195.98(1964.8-2464.91) | 4.37(4.07-4.71) | 204.49(171.39-249.54) | 0.41(0.36-0.48) |
|  | 35 to 39 | 5525.69(4944.64-6110.42) | 9.91(9.27-10.52) | 541.55(465.86-640) | 0.97(0.87-1.1) |
|  | 40 to 44 | 10918.77(10035.58-11807.7) | 19.66(18.85-20.43) | 1055.75(924.38-1221.41) | 1.9(1.74-2.11) |
|  | 45 to 49 | 20270.15(19054.76-21569.63) | 37.48(36.7-38.38) | 2329.04(2085.44-2637.64) | 4.31(4.02-4.69) |
|  | 50 to 54 | 36893.39(34999.66-38708.15) | 69.21(68.29-69.97) | 4812.37(4392.92-5306.23) | 9.03(8.57-9.59) |
|  | 55 to 59 | 60963.08(58061.18-63829.53) | 116.53(115.34-117.65) | 8907.35(8118.64-9751.41) | 17.03(16.13-17.97) |
|  | 60 to 64 | 109753.68(105106.01-114431.84) | 213.57(213.13-214.19) | 23360.34(21635.92-25280.59) | 45.46(43.87-47.32) |
|  | 65 to 69 | 154992.92(147716.87-162384.59) | 336.82(334.47-339.37) | 39767.17(36997.93-42537.2) | 86.42(83.77-88.9) |
|  | 70 to 74 | 197647.48(186923.46-207962.77) | 506.71(498.75-513.39) | 68481.63(64059.94-72713.94) | 175.57(170.92-179.51) |
|  | 75 to 79 | 202337.5(189295.69-213160.76) | 736.82(714.09-750.23) | 78039.9(72006.55-83077.49) | 284.19(271.63-292.39) |
|  | 80 to 84 | 271731.74(240847.24-292810.55) | 1329.11(1222.57-1382.22) | 121511.76(107133.81-131509.96) | 594.35(543.82-620.8) |
|  | 85 to 89 | 323028.45(271012.94-354179.86) | 2534.96(2206.06-2682.65) | 149198.55(125182.72-164345.17) | 1170.83(1018.99-1244.79) |
|  | 90 to 94 | 216720.55(176574.44-238926.86) | 4381.27(3691.61-4677.2) | 100126.57(82696.07-111352.37) | 2024.18(1728.91-2179.82) |
|  | 95 plus | 90024.57(69997.78-101600.05) | 6473.04(5200.74-7076.96) | 42620.04(33481.9-48599.24) | 3064.51(2487.66-3385.18) |

**Supplementary Table 12. Age-specific DALYs of ischemic heart disease and ischemic stroke in Europe, 2023**

| **Location** | | **Ischemic heart disease** | | **Ischemic stroke** | |
| --- | --- | --- | --- | --- | --- |
|  |  | **DALYS number(95%UI)** | **ASDR (95%UI) per 100,000** | **DALYS number(95%UI)** | **ASDR (95%UI) per 100,000** |
| **Europe** | 0 to 4 | 0(0-0) | 0(0-0) | 1029.23(824.92-1259.72) | 2.97(2.47-3.5) |
|  | 5 to 9 | 0(0-0) | 0(0-0) | 2292.67(1664.19-3099.07) | 5.7(4.31-7.41) |
|  | 10 to 14 | 0(0-0) | 0(0-0) | 4028.33(2837.72-5439.88) | 9.41(6.91-12.23) |
|  | 15 to 19 | 7864.42(6706.35-9171.37) | 18.92(16.8-21.25) | 6325.01(4682.94-8281.52) | 15.22(11.73-19.19) |
|  | 20 to 24 | 19456.01(16643.05-22403.03) | 47.2(42-52.39) | 8751.1(6594.36-11285.84) | 21.23(16.64-26.39) |
|  | 25 to 29 | 44991.03(39299.51-51003.96) | 104.41(94.87-114.07) | 13284.2(9921.63-16820.89) | 30.83(23.95-37.62) |
|  | 30 to 34 | 137022.61(122917.63-152881.08) | 272.39(254.81-292.39) | 26857.01(21269.45-33396.12) | 53.39(44.09-63.87) |
|  | 35 to 39 | 314070.07(281447.05-343519.81) | 563.41(527.72-591.55) | 53309.64(44231.98-63956) | 95.63(82.94-110.13) |
|  | 40 to 44 | 562177.63(514585.58-607067.1) | 1012.08(966.4-1050.17) | 86383.35(72969.55-101725.28) | 155.52(137.04-175.98) |
|  | 45 to 49 | 943321.96(884598.29-1006553.72) | 1744.16(1703.82-1791.22) | 151989.99(131904.61-174775.37) | 281.02(254.06-311.02) |
|  | 50 to 54 | 1529320.27(1448033.33-1612589.64) | 2869.03(2825.2-2915.02) | 260708.46(230908.2-294751.8) | 489.09(450.52-532.81) |
|  | 55 to 59 | 2220968.18(2100669.03-2333269.03) | 4245.41(4173.07-4300.76) | 414625.8(369205.93-462015.89) | 792.56(733.44-851.6) |
|  | 60 to 64 | 3419901.24(3259377.44-3584696.6) | 6654.71(6609.17-6709.69) | 849270.65(777864.4-930213.1) | 1652.58(1577.31-1741.14) |
|  | 65 to 69 | 4065241.36(3867689.14-4266421.87) | 8834.37(8757.45-8916.34) | 1188142.63(1087014.23-1298073.25) | 2582.01(2461.28-2712.83) |
|  | 70 to 74 | 4238469.89(4002421.01-4459965.02) | 10866.24(10679.23-11010.21) | 1617621.79(1500758.63-1742785.04) | 4147.13(4004.31-4302.37) |
|  | 75 to 79 | 3482943.54(3252091.08-3681044.07) | 12683.32(12267.98-12955.56) | 1477640.03(1360458.15-1594521.79) | 5380.9(5132.1-5611.97) |
|  | 80 to 84 | 3552310.48(3172768.51-3833593) | 17375.26(16105.32-18096.55) | 1712374.38(1515856.17-1860042.99) | 8375.66(7694.65-8780.37) |
|  | 85 to 89 | 3341828.48(2841644.56-3655167.46) | 26224.94(23131.11-27685.21) | 1643140.99(1399209.4-1806674.58) | 12894.52(11389.63-13684.23) |
|  | 90 to 94 | 1921951.93(1585117.77-2116114.26) | 38854.64(33139.78-41424.75) | 938098.88(776217.45-1037406.35) | 18964.83(16228.24-20308.12) |
|  | 95 plus | 747012.52(585710.38-842586.8) | 53712.44(43517.45-58690.45) | 371806.92(295269.84-420966.06) | 26734.03(21938.13-29322.42) |

Supplementary Figure S1. Mendelian randomization diagnostic plots for fasting glucose and IHD. (A) Scatter plot of SNP–fasting-glucose and SNP–IHD associations with slopes from IVW, MR-Egger, weighted-median, weighted-mode, and simple-mode methods. (B) SNP-level forest plot. (C) Leave-one-out analysis. (D) Funnel plot.

Supplementary Figure S2. Mendelian randomization diagnostic plots for genetic liability to high blood pressure and IHD. (A) Scatter plot of SNP–high-blood-pressure and SNP–IHD associations with slopes from IVW, MR-Egger, weighted-median, weighted-mode, and simple-mode methods. (B) SNP-level forest plot. (C) Leave-one-out analysis. (D) Funnel plot. The exposure corresponds to OpenGWAS ebi-a-GCST90013966 (high blood pressure).

Supplementary Figure S3. Mendelian randomization diagnostic plots for LDL cholesterol and IHD. (A) Scatter plot of SNP–LDL-cholesterol and SNP–IHD associations with slopes from IVW, MR-Egger, weighted-median, weighted-mode, and simple-mode methods. (B) SNP-level forest plot. (C) Leave-one-out analysis. (D) Funnel plot.

Supplementary Figure S4. Mendelian randomization diagnostic plots for fasting glucose and ischemic stroke. (A) Scatter plot of SNP–fasting-glucose and SNP–ischemic-stroke associations with slopes from IVW, MR-Egger, weighted-median, weighted-mode, and simple-mode methods. (B) SNP-level forest plot. (C) Leave-one-out analysis. (D) Funnel plot.

Supplementary Figure S5. Mendelian randomization diagnostic plots for genetic liability to high blood pressure and ischemic stroke. (A) Scatter plot of SNP–high-blood-pressure and SNP–ischemic-stroke associations with slopes from IVW, MR-Egger, weighted-median, weighted-mode, and simple-mode methods. (B) SNP-level forest plot. (C) Leave-one-out analysis. (D) Funnel plot. The exposure corresponds to OpenGWAS ebi-a-GCST90013966 (high blood pressure).

Supplementary Figure S6. Mendelian randomization diagnostic plots for LDL cholesterol and ischemic stroke. (A) Scatter plot of SNP–LDL-cholesterol and SNP–ischemic-stroke associations with slopes from IVW, MR-Egger, weighted-median, weighted-mode, and simple-mode methods. (B) SNP-level forest plot. (C) Leave-one-out analysis. (D) Funnel plot.

**Supplementary Table 13. Outcome-specific GBD risk factors included in the European IHD and IS attribution analysis**

| **GBD risk factor** | **IHD** | **IS** |
| --- | --- | --- |
| Smoking | Included | Included |
| Secondhand smoke | Included | Included |
| Chewing tobacco | — | Included |
| High alcohol use | Included | Included |
| Low physical activity | Included | Included |
| High body-mass index | Included | Included |
| High fasting plasma glucose | Included | Included |
| High systolic blood pressure | Included | Included |
| High LDL cholesterol | Included | Included |
| Kidney dysfunction | Included | Included |
| Particulate matter pollution | Included | Included |
| Lead exposure | Included | Included |
| Low temperature | Included | Included |
| High temperature | Included | Included |
| Diet low in whole grains | Included | Included |
| Diet low in fruits | Included | Included |
| Diet low in vegetables | Included | Included |
| Diet low in legumes | Included | — |
| Diet low in nuts and seeds | Included | — |
| Diet low in fiber | Included | Included |
| Diet low in seafood omega-3 fatty acids | Included | — |
| Diet low in omega-6 polyunsaturated fatty acids | Included | Included |
| Diet high in sodium | Included | Included |
| Diet high in red meat | Included | Included |
| Diet high in processed meat | Included | Included |
| Diet high in sugar-sweetened beverages | Included | Included |
| Diet high in trans fatty acids | Included | — |

Note: Included identifies a risk analyzed for the corresponding outcome in Figure 4. A dash indicates that the risk was outside that outcome-specific profile, not a zero attributable fraction. Risks were selected separately for IHD and IS from the GBD 2023 European regional extraction. GWAS availability affected only MR scope. Risk-specific fractions overlap and should not be summed. IHD, ischemic heart disease; IS, ischemic stroke.

**Supplementary Table 14. GWAS datasets used in the complementary Mendelian randomization analysis.**

| **Role** | **Trait** | **OpenGWAS ID** | **Ancestry** | **Sample size** | **nSNP used** | **Source** |
| --- | --- | --- | --- | --- | --- | --- |
| Exposure | High blood pressure | ebi-a-GCST90013966 | European | 407,746 | 184 (IHD); 201 (IS) | OpenGWAS metadata; Mbatchou et al. [11] |
| Exposure | Fasting glucose | ebi-a-GCST90002232 | European | 200,622 | 65 (IHD); 64 (IS) | Chen et al. [12] |
| Exposure | LDL cholesterol | ebi-a-GCST90002412 | European | 431,167 | 321 (IHD); 339 (IS) | Klimentidis et al. [13] |
| Outcome | Ischemic heart diseases | finn-b-I9_ISCHHEART | European | 30,952 cases; 187,840 controls | — | FinnGen; Kurki et al. [14] |
| Outcome | Ischemic stroke | ebi-a-GCST90018864 | European | 11,929 cases; 472,192 controls | — | Sakaue et al. [15] |

Abbreviations: GWAS, genome-wide association study; IHD, ischemic heart disease; IS, ischemic stroke; LDL, low-density lipoprotein; nSNP, number of single-nucleotide-polymorphism instruments retained after harmonization for the indicated outcome.

Note: High blood pressure denotes the binary OpenGWAS phenotype ebi-a-GCST90013966, as described in manuscript Section 2.6. A dash indicates that an instrument count is not applicable to the outcome dataset row. Reference numbers [11–15] correspond to the manuscript reference list.

**Supplementary Table 15. Cross-country Spearman concordance between IHD and IS age-standardized rates in 2023**

| **Metric** | **n countries** | **Spearman ρ** | **Approximate 95% CI** | **P value** |
| --- | --- | --- | --- | --- |
| ASPR | 44 | 0.681 | 0.482–0.814 | <0.001 |
| ASIR | 44 | 0.729 | 0.552–0.843 | <0.001 |
| ASMR | 44 | 0.814 | 0.682–0.895 | <0.001 |
| ASDR | 43 | 0.820 | 0.690–0.899 | <0.001 |

Note: Regional aggregate rows were excluded. Approximate CIs use Fisher z transformation. One country-level ASDR pair was unavailable in the source table. Abbreviations: IHD, ischemic heart disease; IS, ischemic stroke; ASPR, age-standardized prevalence rate; ASIR, age-standardized incidence rate; ASMR, age-standardized mortality rate; ASDR, age-standardized disability-adjusted life-year rate; CI, confidence interval; n, number of countries; ρ, Spearman rank correlation coefficient.

**Supplementary Figure S7. Detailed Joinpoint trajectories of age-standardized IHD and IS burden in Central, Eastern, and Western Europe, 1990–2023.**

Detailed segment-specific APCs and 95% CIs are reported in Supplementary Tables 5–8.

**Supplementary Figure S8. Age-specific numbers and rates of IHD and IS in Europe, 2023.**

Age-specific data were aggregated for Europe and are presented descriptively; regional age decomposition was not performed.

**References cited in Supplementary Table 14**

11. Mbatchou J, Barnard L, Backman J, et al. Computationally efficient whole-genome regression for quantitative and binary traits. Nat Genet. 2021;53(7):1097–1103.

12. Chen J, Spracklen CN, Marenne G, et al. The trans-ancestral genomic architecture of glycemic traits. Nat Genet. 2021;53(6):840–860.

13. Klimentidis YC, Arora A, Newell M, et al. Phenotypic and genetic characterization of lower LDL cholesterol and increased type 2 diabetes risk in the UK Biobank. Diabetes. 2020;69(10):2194–2205.

14. Kurki MI, Karjalainen J, Palta P, et al. FinnGen provides genetic insights from a well-phenotyped isolated population. Nature. 2023;613:508–518.

15. Sakaue S, Kanai M, Tanigawa Y, et al. A cross-population atlas of genetic associations for 220 human phenotypes. Nat Genet. 2021;53(10):1415–1424.
