# Supplementary material for: Regional Heterogeneity in Ischemic Heart Disease and Ischemic Stroke Burden in Europe, 1990–2023: GBD 2023 and Complementary Mendelian Randomization Evidence
Source: Clin Cardiol. 2026 Oct 23;49(10):e70505. doi: 10.1002/clc.70505 (PMC13691926; doi:10.1002/clc.70505)
Supplement: Supplementary file 2 [file CLC-49-e70505-s002.docx]

| **Item** | **Section** | **How addressed** | **Location** |
| --- | --- | --- | --- |
| 1 | Title/Abstract | MR identified in title and structured abstract. | Title; Abstract |
| 2 | Background | Rationale for complementary MR and distinction from GBD CRA explained. | Introduction paragraphs 2–3 |
| 3 | Objectives | Specific MR objective stated; causal inference framed as conditional on IV assumptions. | Introduction final paragraph |
| 4 | Study design/data sources | GWAS IDs, ancestry, sample size, phenotype, instrument counts, and published source studies are reported. | Methods 2.6; Supplementary Table 14 |
| 5 | Assumptions | Relevance, independence, and exclusion restriction stated explicitly. | Methods 2.7 |
| 6 | Main statistical methods | Instrument thresholds, LD clumping, harmonization, IVW estimator, effect measure, and multiplicity correction described. | Methods 2.7 |
| 7 | Assessment of assumptions | Instrument-strength threshold and pleiotropy-robust alternative estimators/diagnostics described. | Methods 2.7 |
| 8 | Sensitivity analyses | MR-Egger, weighted median, simple/weighted mode, SNP forest, leave-one-out, funnel plots. | Methods 2.7; Supplementary Figures S1–S6 |
| 9 | Software/pre-registration | R 4.4.1 and MR packages stated; study was not described as preregistered because no preregistration evidence was available. | Methods 2.7 |
| 10 | Descriptive data | GWAS sample sizes, ancestry, case/control numbers where available, and nSNP reported. Potential overlap limitation stated. | Supplementary Table 14; Methods 2.7 |
| 11 | Main results | IVW ORs, 95% CIs, P values, nSNP, and Bonferroni interpretation reported. | Results 3.6; Figure 5 |
| 12 | Assessment of assumptions | Available sensitivity evidence is presented through alternative MR estimators and graphical diagnostics; numerical heterogeneity/intercept statistics were not available for reporting. | Results 3.6; Supplementary Figures S1–S6 |
| 13 | Additional/sensitivity analyses | Alternative estimators and leave-one-out/funnel diagnostics provided. | Supplementary Figures S1–S6 |
| 14 | Key results | MR results summarized with reference to complementary-study objective. | Discussion paragraph 1 |
| 15 | Limitations | IV assumptions, potential pleiotropy, ancestry/sample overlap, limited exposures, overall-IS phenotype, and limitations of the available sensitivity diagnostics are discussed. | Discussion limitations paragraph |
| 16 | Interpretation | Causal language qualified; phenotype differences and IS subtype heterogeneity discussed. | Discussion paragraphs 5–6 |
| 17 | Generalizability | Generalizability is discussed with respect to predominantly European-ancestry GWAS and the use of overall rather than subtype-specific ischemic stroke. | Discussion; Limitations |
| 18 | Funding | Funding statement provided. | Declarations |
| 19 | Data sharing | GBD and OpenGWAS access information and GWAS IDs provided. | Data availability |
| 20 | Conflicts of interest | Competing-interests statement provided. | Declarations |
